# Whole-brain modelling identifies distinct but convergent paths to unconsciousness in anaesthesia and disorders of consciousness

**DOI:** 10.1038/s42003-022-03330-y

**Published:** 2022-04-20

**Authors:** Andrea I. Luppi, Pedro A. M. Mediano, Fernando E. Rosas, Judith Allanson, John D. Pickard, Guy B. Williams, Michael M. Craig, Paola Finoia, Alexander R. D. Peattie, Peter Coppola, Adrian M. Owen, Lorina Naci, David K. Menon, Daniel Bor, Emmanuel A. Stamatakis

**Affiliations:** 1 Division of Anaesthesia, School of Clinical Medicine, University of Cambridge, Cambridge, UK; 2 Department of Clinical Neurosciences, University of Cambridge, Cambridge, UK; 3 Leverhulme Centre for the Future of Intelligence, University of Cambridge, Cambridge, UK; 4 The Alan Turing Institute, London, UK; 5 Department of Psychology, University of Cambridge, Cambridge, UK; 6 Department of Psychology, Queen Mary University of London, London, UK; 7 Center for Psychedelic Research, Department of Brain Science, Imperial College London, London, UK; 8 Data Science Institute, Imperial College London, London, UK; 9 Centre for Complexity Science, Imperial College London, London, UK; 10 Department of Neurosciences, Cambridge University Hospitals NHS Foundation, Addenbrooke’s Hospital, Cambridge, UK; 11 Division of Neurosurgery, School of Clinical Medicine, University of Cambridge, Cambridge, UK; 12 Wolfson Brain Imaging Centre, University of Cambridge, Cambridge, UK; 13 The Brain and Mind Institute, University of Western Ontario, London, ON Canada; 14 Trinity College Institute of Neuroscience, Trinity College Dublin, Dublin, Ireland

**Keywords:** Biophysical models, Dynamical systems, Network models, Consciousness

## Abstract

The human brain entertains rich spatiotemporal dynamics, which are drastically reconfigured when consciousness is lost due to anaesthesia or disorders of consciousness (DOC). Here, we sought to identify the neurobiological mechanisms that explain how transient pharmacological intervention and chronic neuroanatomical injury can lead to common reconfigurations of neural activity. We developed and systematically perturbed a neurobiologically realistic model of whole-brain haemodynamic signals. By incorporating PET data about the cortical distribution of GABA receptors, our computational model reveals a key role of spatially-specific local inhibition for reproducing the functional MRI activity observed during anaesthesia with the GABA-ergic agent propofol. Additionally, incorporating diffusion MRI data obtained from DOC patients reveals that the dynamics that characterise loss of consciousness can also emerge from randomised neuroanatomical connectivity. Our results generalise between anaesthesia and DOC datasets, demonstrating how increased inhibition and connectome perturbation represent distinct neurobiological paths towards the characteristic activity of the unconscious brain.

## Abstract (web summary)

Perturbations in a large-scale whole-brain model suggest that anesthesia and injury may be imparting functionally similar effects in terms of brain dynamics.

## Introduction

The human brain generates a dynamically changing repertoire of neural activity, supporting its rich variety of conscious experiences and cognitive functions^1–15^. A central challenge of contemporary neuroscience is the quest to understand how the neurobiology and function of the human brain give rise to such rich conscious experience^16,17^. One way to address this question is to identify changes in brain function that accompany changes in conscious state^18^. Recently, increased focus on brain dynamics^2,19–25^ has enabled substantial progress on this question^18,26–35^.

However, the brain is a paradigmatic example of a complex system^36^, and different perturbations of its precise functioning can serve as a path towards loss of consciousness. Examples of such perturbations range from transient pharmacological (general anaesthetic) interventions having widespread effects on neuromodulation^37–40^, to chronic disorders of consciousness arising from injuries of diverse location and extent, often including changes to the physical connectivity between brain regions^41–58^. This similarity of outcomes and neural signatures^18,26–30^ despite arising from radically different causes, begs the question: How can (transient) pharmacological and (chronic) structural perturbations converge to similar effects on dynamic brain activity, and the corresponding state of unconsciousness?

Here, we sought to obtain mechanistic insights into this fundamental question by employing whole-brain computational modelling. Neuropsychological studies in human patients and experimental lesions in animal models have provided invaluable insights about brain organisation, function and dysfunction^59–62^. Whole-brain computational models can be systematically and reversibly manipulated in ways that are still beyond the capabilities of experimental research, whether in humans or animals^63,64^. Therefore, in-silico whole-brain models^23,65,66^ are uniquely suited to investigate how different neurobiological perturbations can induce similar alterations of brain activity^23,63,66–78^, including recent successful applications to the study of consciousness with oscillator-based models (Hopf)^79–85^ or models based on statistical mechanics (Ising)^86–88^.

Crucially, recent work has demonstrated that more detailed biophysical models that incorporate neurophysiologically realistic information about excitation, inhibition and neuromodulation – so-called Dynamic Mean Field (DMF) models – can provide insights about pharmacologically-induced changes in macroscale fMRI haemodynamics, in terms of the underlying neurobiology^89–91^. Such neurobiologically realistic computational models provide a principled way to bridge across scales, relating the macroscale neural dynamics of fMRI to the microscale neurophysiological mechanisms from which they emerge^63,92^. However, to date no studies have harnessed the power of DMF models to provide neurobiologically realistic accounts of pharmacological and chronic loss of consciousness.

Here, we leveraged a neurobiologically realistic DMF model informed by multimodal neuroimaging including empirical brain activity from functional MRI, anatomical connectivity obtained from diffusion MRI, and GABA-A receptor density estimated from positron emission tomography (PET). We used this modelling approach to simulate the empirical fMRI macroscale brain activity observed in the same *n* = 16 subjects during wakefulness and during loss of consciousness induced by the intravenous anaesthetic, propofol. We also studied the fMRI activity of a cohort (*n* = 21) of patients suffering from chronic disorders of consciousness (DOC) as a result of severe brain injury (traumatic or anoxic), comparing them with a group of *n* = 20 healthy controls. By subjecting the models to virtual anaesthesia (local modulation of inhibitory gain based on empirical GABA-A receptor distribution) and virtual DOC (alteration of the model’s structural connectome), we sought to identify the neurobiological mechanisms underlying a fundamental question of modern neuroscience: how can transient perturbations of neurotransmission and chronic lesions to the structural connectome, both give rise to unconsciousness and its characteristic similar haemodynamic signatures ^18,26,27,29,93^?

## Results

**Fig. 1:**
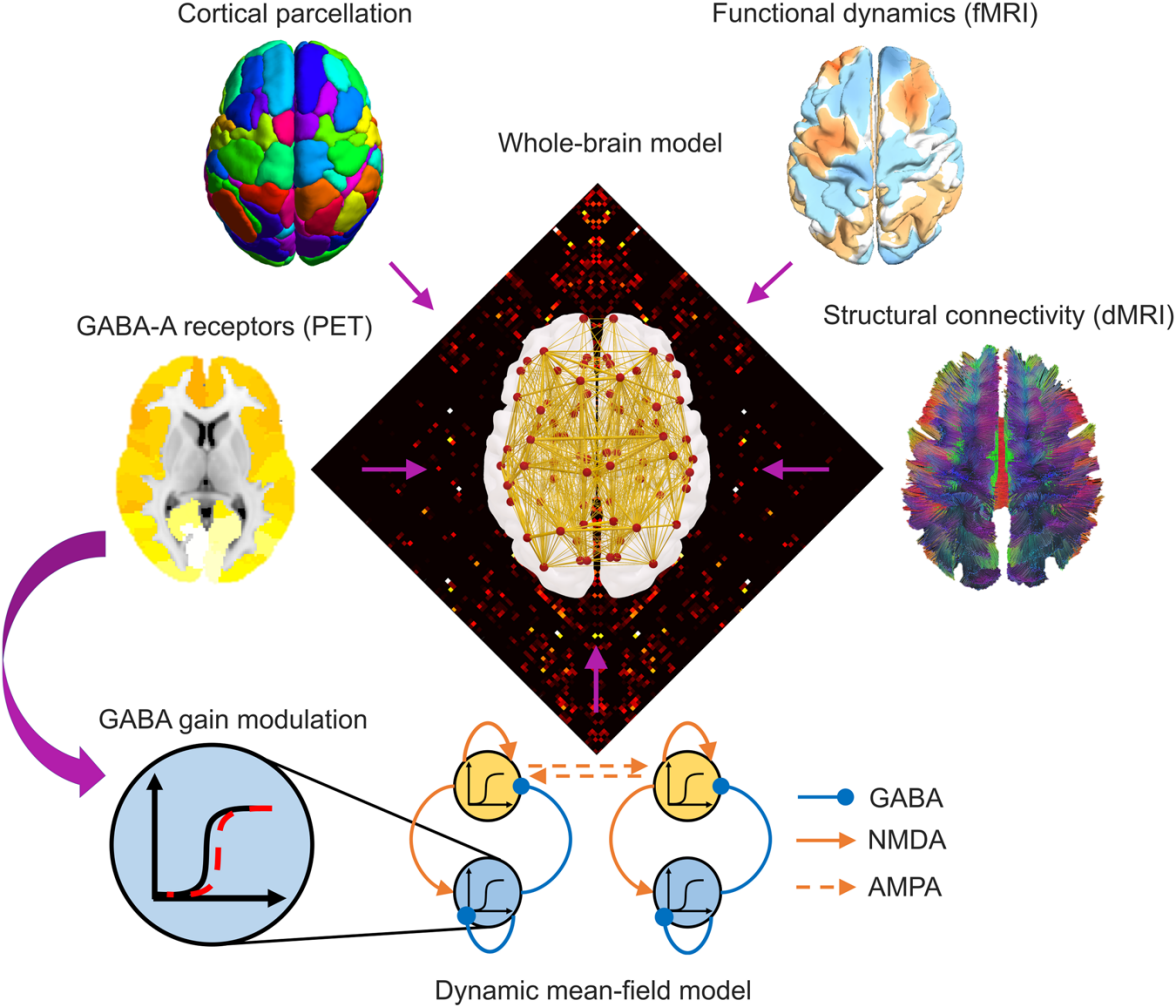
Overview of whole-brain computational model incorporating multimodal neuroimaging data. Based on a cortical parcellation with 68 regions of interest, each node (cortical region) is modelled through a neurophysiologically realistic biophysical model incorporating excitatory (NMDA) as well as inhibitory (GABA) synaptic dynamics. Nodes are connected by structural connectivity (from diffusion MRI) and the model’s simulated BOLD signals are fitted to simulate empirical BOLD signal patterns (from functional MRI). Neurotransmitter information from PET can also be added in the model as modulating the local neuronal gain^89^.

We employed a neurobiologically realistic dynamic mean-field (Fig. 1) model to investigate how perturbations of neurotransmission and lesions to brain connectivity can both give rise to the characteristic spatiotemporal patterns of brain activity observed during loss of consciousness. The DMF model reduces the intricate dynamics of individual neurons to a set of coupled differential equations which approximate the detailed microscale neural properties of spiking neurons (incorporating realistic aspects of neurophysiology such as synaptic dynamics and membrane potential)^94^ via a mean-field reduction^19,22,25,95,96^. Specifically, cortical regions are represented as macroscopic neural fields, whose local dynamics are coupled together by a network of anatomical connections^19,22,25^. An additional biophysical haemodynamic model can then be used to turn the DMF model’s activity into a realistic simulator of BOLD signals^97^.

Following previous DMF modelling work^89^, we evaluate the quality of fit in terms of the Kolmogorov-Smirnov distance between the distributions of real and simulated functional connectivity dynamics (FCD), corresponding to the patterns of inter-temporal correlations between sliding windows of functional connectivity, thereby taking into account both spatial and temporal aspects of haemodynamic activity. We followed this procedure for each of our two datasets (Supplementary Figure 1 and Methods): for the propofol dataset we optimised the model to fit the functional connectivity dynamics of empirical fMRI data acquired during the awake scan, and for the DOC dataset we optimised the model to fit the FCD observed in the healthy controls. These two calibrated DMF models - with their corresponding global coupling values fitted to the BOLD signals of the conscious brain for each of our two datasets - constitute the starting point for our investigations.

### Inhibitory modulation from GABA-A receptor distribution reveals a shared mechanism for loss of consciousness

Propofol is a potent agonist of inhibitory GABA-A receptors^98,99^. The effects of propofol anaesthesia on the brain were therefore modelled by capitalising on the recently built whole-brain map of GABA-A regional receptor density, generated on the basis of benzodiazepine receptor (BZR) density measured from [^11^C]flumazenil Positron Emission Tomography (PET; see Methods)^100^. Incorporating this information in the DMF model allowed us to evaluate the extent to which the dynamics of the anaesthetised brain can be explained in terms of propofol-induced alterations in the detailed balance of local excitation and inhibition.

In seminal previous work, Deco and colleagues^89^ modelled the effects of the serotonergic drug LSD by locally modulating the neuronal gain of each excitatory population in the model according to the empirical distribution of 5HT-2A receptors across brain regions^89^. Inspired by their approach, here we demonstrate that the influence of regional GABA-A receptor density on functional dynamics can be modelled using a DMF model informed by regional GABA-A receptor density.

**Fig. 2:**
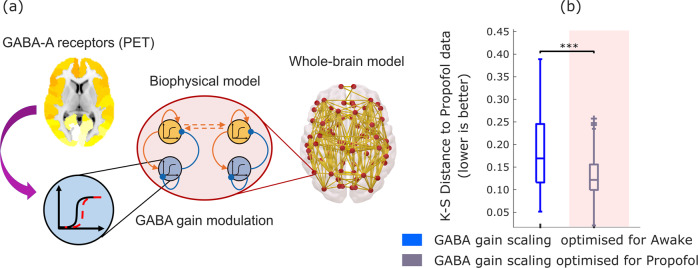
Modulation of inhibitory gain by empirical GABA-A receptor density improves model fit to propofol dynamics. **a** The inhibitory gain of each node in the balanced DMF model is modulated by the regional density of GABA-A receptors, estimated from PET. **b** Box-plots show the model fit for *n* = 100 simulations, quantified as the KS-distance (lower is better) to the functional connectivity dynamics (FCD) derived from the propofol (anaesthetised) condition, using a value of gain for inhibitory scaling *s*_*I*_ derived from calibrating the model with respect to either the awake (blue) or propofol (grey on shaded background) conditions. Middle line: median; box limits, upper and lower quartiles; whiskers, 1.5x inter-quartile range; “+” symbol indicates outliers; ***p < 0.001 from *t*-test. Source data are provided in Supplementary Data 1. We replicated this result using an alternative version of the KS-distance, which in addition to the distribution of FCD values also takes into account the temporal lag between them (Supplementary Fig. 2).

The strategy followed by Deco and colleagues^89^ was to first calibrate the model on awake data to obtain a global coupling value (see Methods), and then fit a secondary inhibitory parameter separately on awake and post-propofol (anaesthetised) data. Our approach follows Deco’s but differs in one key respect: given the inhibitory nature of GABA, we modulated the inhibitory (rather than excitatory) local gain (note that the excitatory and inhibitory populations within each region in the biophysical model are mutually and recursively coupled, and hence both excitation and inhibition are eventually affected by this procedure) (Fig. 2a). To do so, we introduced an inhibitory gain scaling parameter in the model, denoted by *s*_*I*_. This parameter allowed us to scale the inhibitory gain at each region according to the empirical local density of GABA-A receptors, as quantified based on PET-derived maps of receptor density^100^.

This procedure allowed us to ask whether adjusting the value of inhibitory gain *s*_*I*_ according to local GABA-A receptor density would allow the model to simulate the spatiotemporal patterns of haemodynamic activity that characterise acute propofol-induced unconsciousness. A positive answer to this question would implicate regional GABA-ergic inhibition as a neurobiological mechanism behind the action of propofol (a known GABA-ergic agonist) in inducing the characteristic macroscale activity patterns observed during loss of consciousness due to propofol anaesthesia^18,26,28,29^.

To address this issue, we studied whether some appropriate value of *s*_*I*_ (which scales the gain related to the local GABA-A receptor density) would improve the model’s ability to simulate the dynamics of deep propofol anaesthesia. For this purpose, we used the previously calibrated DMF model to generate simulations for each value of *s*_*I*_ between 0 (corresponding to the model without local GABA inhibitory modulation) and 1, in increments of 0.02. Then, for each value of *s*_*I*_, we computed the KS distance between the model’s simulated functional connectivity dynamics, and the empirical FCD observed in the awake and in the anaesthetised subjects, respectively. Separately for each condition, the optimal value of *s*_*I*_, was then identified as the value that resulted in the minimum mean KS distance between empirical and simulated FCD (across *n* = 10 simulations for each value of *s*_*I*_).

Having completed the fitting procedure for our models, we then proceeded to analyse the models’ performance. To this end, we generated *n* = 100 simulations from each of the propofol-fitting and awake-fitting models. For both models, we then computed the KS distance between each simulation, and the empirical FCD observed during wakefulness, and during anaesthesia. This provided us with a way to quantify the ability of each model (in terms of goodness of fit, i.e., low KS distance) to simulate the empirical patterns of spatiotemporal brain activity observed during wakefulness, and the empirical patterns of spatiotemporal brain activity observed during propofol-induced loss of consciousness.

Our results indicate that the DMF model’s ability to simulate the empirical FCD observed during propofol-induced anaesthesia can be significantly improved (lower KS-distance) by increasing the inhibitory gain scaling from the value that best reproduces the awake functional connectivity dynamics (*s*_*I*_ = 0.02) to a higher value (*s*_*I*_ = 0.52) (Fig. 2b and Supplementary Table 1). In other words, the modulation of inhibition in accordance with the empirical distribution of GABA-A receptors across brain regions, makes the model capable of switching between simulating awake or anaesthetised brain activity. Since propofol is a well-known GABA-ergic agonist, these results confirm that taking into account GABA agonism (local modulation of inhibitory gain by regional GABA-A receptor density) is sufficient to recapitulate the known effects of the GABA-ergic agent propofol on empirical brain activity patterns, leading to dynamics that are known to characterise the state of unconsciousness.

**Fig. 3:**
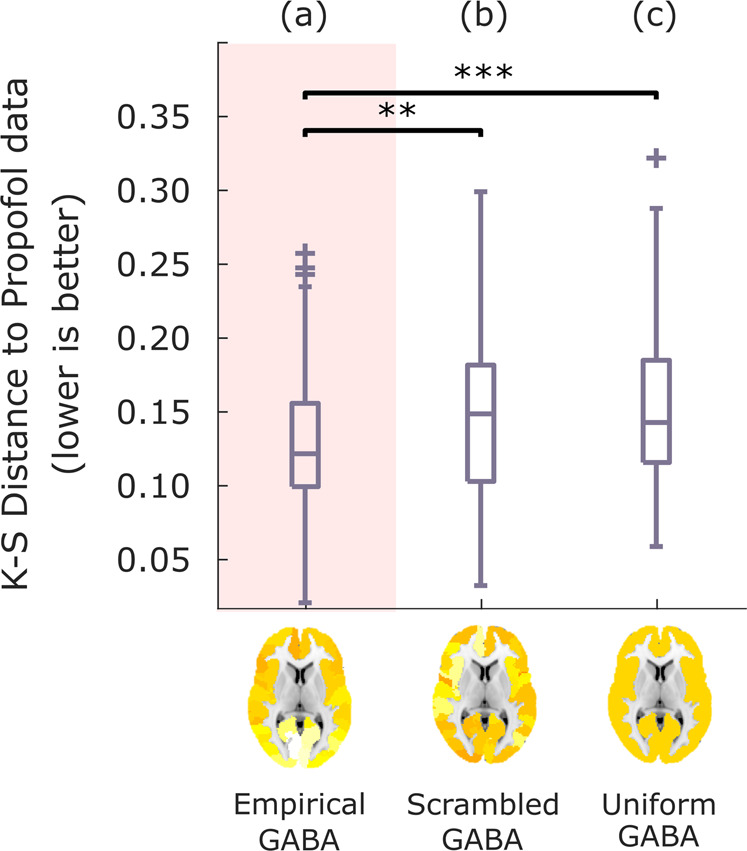
Modulation of inhibitory gain by reshuffled or uniform GABA-A receptor density. After identifying the value of *s*_*I*_ that leads to the best fit with the empirical propofol data when modulating the inhibitory gain of each node in the balanced DMF model according to the empirical distribution of GABA-A receptors (**a**), the simulation is repeated after randomly reshuffling the regional receptor densities across the cortex (**b**), or setting them all to a uniform value (mean of the empirical distribution) (**c**). Box-plots show the model fit to the propofol data for *n* = 100 simulations, quantified as KS-distance (lower is better) between simulated and empirical FCD, for each variant of the model. Middle line: median; box limits, upper and lower quartiles; whiskers, 1.5x inter-quartile range; “+” symbol indicates outliers; **p < 0.01; ***p < 0.001 from *t*-test. Source data are provided in Supplementary Data 1. We replicated this result using an alternative version of the KS-distance, which in addition to the distribution of FCD values also takes into account the temporal lag between them (Supplementary Fig. 2).

Crucially, we also confirmed that the improved fit to anaesthetised dynamics is not merely the result of increasing overall inhibition in the model: rather, regional information about the distribution of GABA receptor density plays a key role in the model’s improved fit. To demonstrate this point, we show that the results are not replicated if the PET-derived regional distribution of GABA-A receptor density is reshuffled across regions while preserving spatial autocorrelation (Methods) (Fig. 3a, b), or if uniform values are used for each region (i.e., by setting all regions to have a value equal to the mean of the distribution; Fig. 3c). In both cases, the model’s ability to fit anaesthetised dynamics is significantly impaired compared with the model using the empirical distribution of GABA-A receptors obtained from in vivo PET imaging (Fig. 3 and Supplementary Table 1). Therefore, our results show that the specific regional distribution of GABA-A receptors across the cortex plays a key role in generating the spatiotemporal patterns of brain activity characteristic of unconsciousness induced by propofol administration.

### Simulated brain injury induces unconscious-like dynamics

Whole-brain computational models provide a unique tool to understand the effects of connectome alterations on macroscale brain activity^73,74,86^. We developed a procedure to probe which of two conditions is more compatible with a given perturbation of the connectome, in terms of the connectome’s capacity to support the corresponding brain activity. We term this procedure, Connectome Replacement Analysis. The procedure involves (i) calibrating the model based on the healthy connectome; (ii) evaluating the relative suitability of this healthy calibrated model to reproduce the empirical activity patterns of the healthy conscious brain, versus the empirical activity patterns of our condition of interest (here, DOC patients), in terms of the difference between the KS-distance to the respective empirical FCDs; (iii) replacing the underlying healthy connectome of the initial calibrated model, with a perturbed connectome, and generating new simulated haemodynamic signals; (iv) re-evaluating the relative difference in KS-distance to each condition (healthy and DOC) for the new simulated brain activity.

**Fig. 4:**
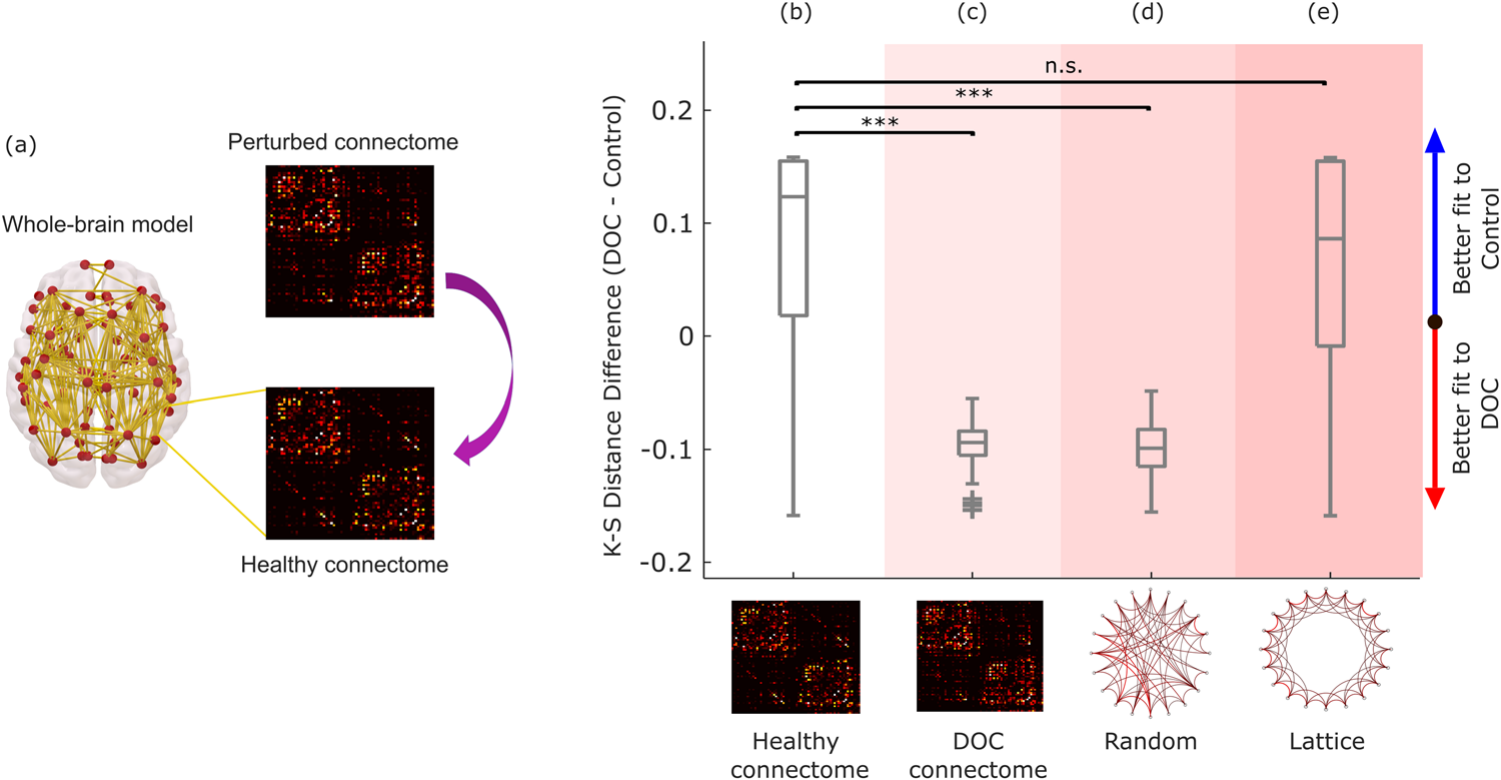
Connectome replacement analysis with DOC connectome. **a** The original healthy connectome of the model is replaced with the group-average connectome obtained from diffusion MRI of *n* = 21 DOC patients, and the resulting model is used to generate *n* = 100 simulations. **b**–**e** Box-plots show the difference in model fit (KS-distance) between the two conditions (fit to DOC patients’ data minus fit to healthy controls’ data, over *n* = 100 simulations), for the initial model calibrated based on the healthy connectome (**b**), and after replacing the model’s initial connectome with either the DOC patients’ empirical consensus connectome (**c**), or after rewiring the initial connectome into a random network (**d**), or into a regular (lattice) network (**e**). Middle line: median; box limits, upper and lower quartiles; whiskers, 1.5x inter-quartile range; “+” symbol indicates outliers; ***p < 0.001; n.s. Not significant (*p* > 0.05) from *t*-test. Source data are provided in Supplementary Data 2. We replicated this result using an alternative version of the KS-distance, which in addition to the distribution of FCD values also takes into account the temporal lag between them (Supplementary Fig. 3).

Leveraging this capability, we subjected the DMF model to the virtual equivalent of severe brain injury: namely, we replaced the underlying connectivity matrix governing the long-range interactions between brain regions with a consensus connectome^101^ obtained from diffusion-weighted imaging of *n* = 21 patients with chronic DOC due to severe brain injury (Fig. 4a). This procedure imparts the model with effects akin to what severe brain injury does on anatomical connectivity. This virtual DOC provides a way to isolate the effects over brain dynamics of connectivity disruptions that result in loss of human consciousness.

Note that such substantial perturbations are naturally expected to deteriorate the model’s ability to replicate empirical brain activity (i.e., increasing the KS distance, corresponding to decreased goodness-of-fit): the initial calibrated model was optimised with biophysical parameters pertaining to healthy brains, and using a healthy connectome. However, what matters for this analysis is not the absolute value of the fit, but rather its relative difference between the two conditions. Specifically, our hypothesis was that the haemodynamic activity generated by the model with DOC connectome should be more similar (lower KS distance, indicating a better fit) to the empirical dynamics of DOC patients’ brains, than to the dynamics of conscious, healthy brains.

Our results supported these predictions. The calibrated model based on the healthy connectome exhibited a better fit for the dynamic activity patterns of the healthy brain than for the DOC patients’ brain activity (positive difference in KS-distance; Fig. 4b and Supplementary Table 2). This pattern was reversed upon replacing the healthy connectome with the consensus connectome^101^ obtained from DOC patients’ DTI data, such that the difference in KS-distance to the two conditions became negative, indicating on average a better fit to the empirical brain activity of DOC patients than healthy controls (Fig. 4c and Supplementary Table 2).

This observation supports our hypothesis, demonstrating that unconscious brain dynamics are more compatible with the DOC connectome than conscious dynamics; below, we also demonstrate that this result is not specific to the chronic unconsciousness that characterises disorders of consciousness, but rather it generalises to the transient unconsciousness caused by propofol anaesthesia, too. We also replicated this result when constructing the DOC “consensus connectome” after excluding the *n* = 6 DOC patients whose diffusion-weighted data were acquired with a different protocol (see Methods), thereby excluding this potential confound as an explanation for our results (Supplementary Fig. 4).

Remarkably, these results could be replicated by replacing the original healthy structural connectome with a randomised version having the same average connectivity^102^. After perturbation, the model’s fit to DOC patients’ empirical brain activity became better than the model’s fit to the spatiotemporal activity of the conscious brain (Fig. 4d and Supplementary Table 2), suggesting that the dynamics underlying unconsciousness are more compatible with a randomised connectome than the dynamics underlying the activity of the conscious brain. In contrast, this effect could not be observed when the original connectome was rewired into a regular (lattice) network, indicating that not just any perturbation of the connectome is suitable to improve the model’s fit to unconscious brain activity vis-à-vis conscious brain activity. Together, these results suggest that DOC dynamics are more compatible with an unstructured connectome.

### Generalisation across datasets

Having identified the role of GABA-mediated inhibition for propofol anaesthesia, we next sought to determine to what extent inhibition can also explain the dynamics of unconsciousness arising from severe brain injury. Our rationale was that, even though these patients have not been exposed to GABA-ergic agents but rather owe their condition to severe brain injury, recent evidence suggests similarities of dynamic spatiotemporal patterns of brain activity during anaesthesia and disorders of consciousness^18,26,27,30,103^. A positive answer to this question would further implicate a change in the excitation-inhibition balance, not just in the generation of brain activity pertaining to propofol anaesthesia, but more broadly as a general mechanism responsible for the characteristic dynamics of unconscious states - whether due to anaesthesia or brain injury.

**Fig. 5:**
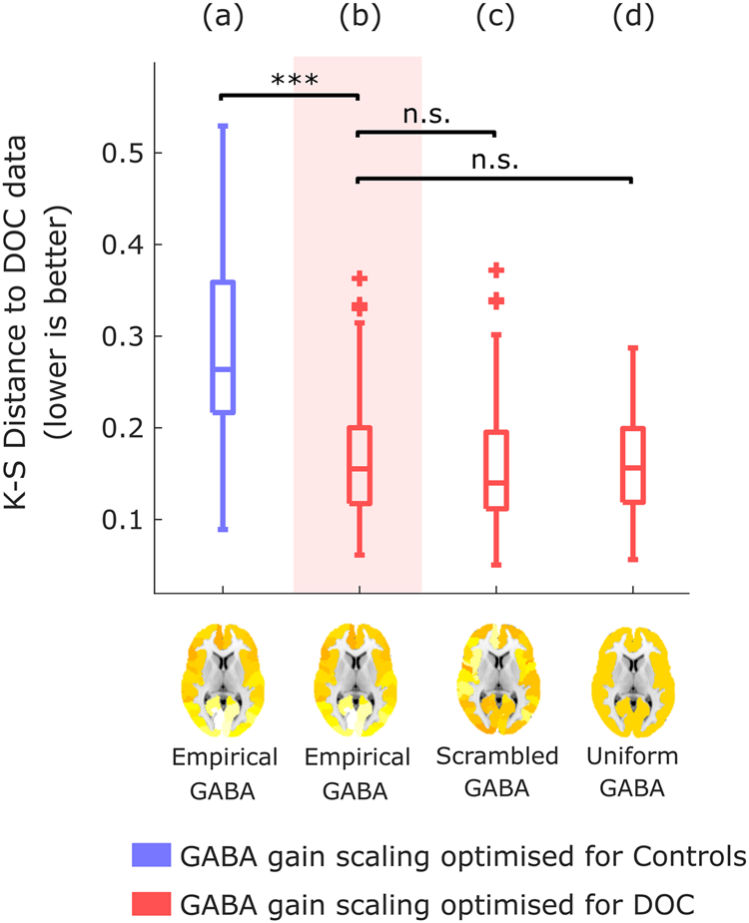
Modulation of inhibitory gain by empirical GABA-A receptor density improves model fit to DOC brain dynamics. Box-plots show the KS-distance (lower is better) to the empirical brain activity of DOC patients, for *n* = 100 simulations of a model that (**a**) is informed by empirical GABA-A regional density, using the value of gain for inhibitory scaling *s*_*I*_ derived from calibrating the model with respect to healthy controls’ empirical brain activity (blue); (**b**) is informed by empirical GABA-A regional density, using the value of gain for inhibitory scaling *s*_*I*_ that provides the best fit to the DOC patients’ empirical brain activity (red); (**c**) same as (**b**), but the regional receptor densities are randomly reshuffled across the cortex; (**d**) same as (**b**), but the receptor densities are all set to a uniform value (the mean of the empirical distribution). Middle line: median; box limits, upper and lower quartiles; whiskers, 1.5x inter-quartile range; “+” symbol indicates outliers; ***p < 0.001. n.s. Not significant (*p* > 0.05) from t-test. Source data are provided in Supplementary Data 3.

Therefore, we followed the same virtual anaesthesia procedure with empirical data from healthy controls and DOC patients (note that this analysis did not involve a direct comparison with the data from the Ontario dataset, neither for the awake nor for the propofol conditions). Intriguingly, we observed analogous results: local modulation of inhibitory gain based on GABA-A receptor density (optimal *s*_*I*_ = 0.4) allowed the model to substantially improve its fit to DOC patients’ brain activity, compared with a model incorporating the same information about receptor distribution, but whose inhibitory gain scaling *s*_*I*_ was optimised to fit the controls’ brain activity (*s*_*I*_ = 0.02) (Fig. 5a, b and Supplementary Table 3). However, in contrast with propofol anaesthesia, the improvements were also observed when the regional receptor map was scrambled, or replaced by a uniform map, such that no significant difference was observed between these latter two models, and the model incorporating the empirical distribution of GABA-A receptors obtained from PET (Fig. 5c, d and Supplementary Table 3). Thus, these results suggest that whereas propofol anaesthesia depends on the specific distribution of GABA-A receptors across the cortex, indicating that these receptors are mediating the effects of propofol, the characteristic dynamics of DOCs are less selective, and appear to correspond to a non-specific increase in global inhibition.

**Fig. 6:**
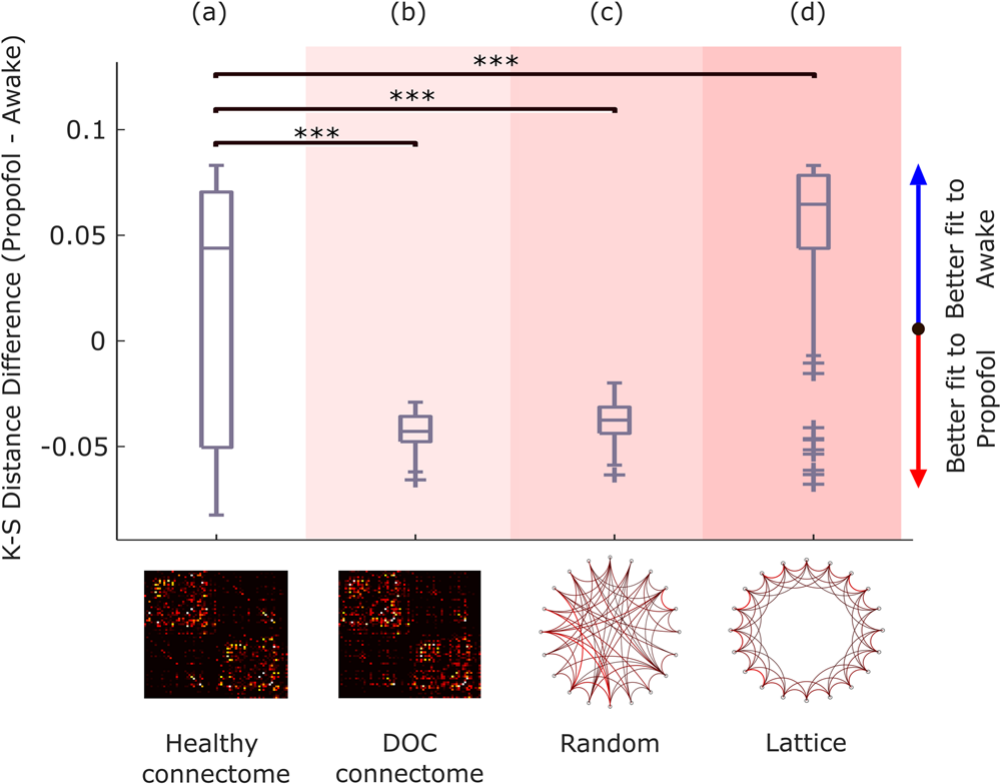
Connectome replacement analysis with DOC connectome generalises to propofol anaesthesia. Box-plots show the difference in model fit (KS-distance) between the two conditions (fit to propofol data minus fit to awake data, over *n* = 100 simulations), for the initial model calibrated based on the healthy connectome (**a**), and after replacing the model’s initial connectome with either the DOC patients’ empirical consensus connectome (**b**), or after rewiring the initial connectome into a random network (**c**), or into a regular (lattice) network (**d**). Middle line: median; box limits, upper and lower quartiles; whiskers, 1.5x inter-quartile range; “+” symbol indicates outliers; ***p < 0.001 from *t*-test. Source data are provided in Supplementary Data 4.

Additionally, if propofol and severe injury are different ways by which the human brain can be pushed towards unconsciousness, then inducing a virtual DOC via connectome replacement should also lead to a model that is better able to simulate the characteristic spatiotemporal activity patterns of an anaesthetised brain, than an awake brain - thereby recapitulating what we previously observed with the macroscale brain activity of DOC patients. Remarkably, results show that - as previously observed with DOC patients -simulated functional connectivity dynamics generated from a model using the DOC connectome are more compatible (lower relative KS distance) with the functional connectivity dynamics of propofol anaesthesia than with the FCD of awake subjects’ brains (Fig. 6a, b and Supplementary Table 4).

Furthermore, the importance of the topology of the perturbed connectome was also observed for brain activity under propofol, with randomisation of the connectome similarly reversing the relative difference in the model’s ability to fit the FCD of conscious brains (awake) vis-à-vis unconscious brains (propofol) in favour of the latter (Fig. 6c and Supplementary Table 4). Conversely, the opposite effect was observed when the original connectome was replaced with a regular (lattice) network, which resulted in a further significant deterioration in the model’s ability to reproduce the FCD of the anaesthetised brain vis-à-vis the awake brain (Fig. 6d and Supplementary Table 4).

These findings generalise our DOC results to propofol anaesthesia, indicating that the DOC connectome is not only more compatible with the macroscale spatiotemporal patterns of DOC patients’ brain activity than with the brain activity of healthy individuals. Rather, the generalisation to propofol anaesthesia suggests that the DOC connectome may be more compatible with unconscious dynamics in general: whether arising from brain injury or pharmacological intervention.

### Alternative approaches

We also considered alternative methodological approaches to complement our main analyses. Pertaining to modelling the propofol data, although for our main analyses we followed previous publications in employing a regionally homogeneous value of the gain scaling parameter *s*_*I*_^89^, we considered whether this value may also vary in a regionally-specific manner - in addition to the regional heterogeneity that we already incorporate in our model by taking into account the empirically-derived regional GABA receptor density. We reasoned that, if there is regional variability in inhibitory gain scaling (independent of the local density of receptors), a plausible account for this phenomenon may be the regional prevalence of specific types of inhibitory interneurons. Therefore, in addition to regional GABA-A receptor density from PET, we added regional variability in *s*_*I*_ values in proportion to empirical regional values of different types of interneurons. We obtained these maps from the Allen Institute for Brain Science transcriptomic dataset, parcellated using standard pipelines from the *abagen* toolbox:^104^ somatostatin-positive (SST + ), parvalbumin-positive (PVALB + ) and vasoactive intestinal peptide-positive (VIP + ) ones, which together account for the majority of cortical interneurons. This analysis did not indicate significant improvements in the ability of the model to fit the propofol data, compared with our main model incorporating regionally heterogeneous GABA-A receptor densities but homogeneous *s*_*I*_ (Supplementary Fig. 5). Although a computationally intensive joint optimisation of both regionally variable parameters may yield additional insights, our results show that taking into account the empirical heterogeneity of regional GABA-A receptor densities is already sufficient to improve the model’s ability to fit propofol data - which was our goal.

Pertaining to modelling the DOC data, insight about the effects of perturbations on a dynamical system can also be obtained by studying the system’s Jacobian, which takes into account both the system’s dynamics and its underlying network structure (here, the different connectomes: healthy, DOC, random and lattice), and whose eigenspectrum provides information about stability in the vicinity of fixed points in the system’s dynamics^105^. In Supplementary Note 1, we show that the real part of the eigenvalues of the reconstructed Jacobians from the healthy connectome exhibits the least similarity (correlation) with the eigenvalues obtained from perturbed connectomes (DOC, random and lattice), which are more highly correlated with each other (Supplementary Fig. 6). While our main results also clearly point to a similarity between the effects of random and DOC connectomes on simulated brain activity, our computational modelling suggests that their effects differ from those of a regular (lattice) network (Fig. 4). Thus, eigenspectrum analysis of the reconstructed system Jacobian and comparison of simulated versus empirical functional connectivity dynamics point to complementary insights that can be derived from these different methodologies.

## Discussion

Here, we sought to identify neurobiological mechanisms that are capable of explaining how highly dissimilar causes - such as transient perturbations of neurotransmission versus chronic lesions to brain anatomy and connectivity - can give rise to loss of consciousness and its characteristic dynamic patterns of brain activity^63^. To this end, we employed a whole-brain Dynamic Mean Field model that simulates the macroscale functional haemodynamics of the human brain by means of neurobiologically realistic biophysical modelling, which integrates empirical spatiotemporal patterns from functional MRI, anatomical connectivity obtained from diffusion MRI, and neurotransmitter receptor density estimated from Positron Emission Tomography^89–91^. Our results demonstrate fundamental similarities, not just between the macroscale dynamic patterns of brain activity that characterise anaesthesia and disorders of consciousness^18,26–30^ but also between the neurobiological mechanisms from which they can arise - despite the fact that anaesthesia is a transient pharmacological intervention and DOCs are the result of permanent neuroanatomical injury. Both disorders of consciousness and propofol anaesthesia were shown to arise from neurobiological mechanisms that are functionally equivalent to connectome randomisation, and both involve increased perturbed excitation-inhibition balance, as indicated by incorporating into the model information about regional GABA-A receptor density estimated from PET

The effect of inhibition was assessed by enriching the DMF model, modulating the neuronal gain of each inhibitory population according to the empirical density of GABA-A receptors across cortical regions, quantified using in-vivo PET^100^. Our results demonstrate that GABA-mediated inhibition plays a mechanistic role in the emergence of the characteristic macroscale dynamic neural activity observed during propofol-induced unconsciousness. These results align with neurophysiological evidence indicating that propofol is primarily a GABA-A receptor agonist^98,99^. Indeed, our results further indicate that propofol anaesthesia is crucially dependent on the specific regional distribution of GABA-A receptors across the cortex, since neither reshuffling this distribution across regions nor setting all regions to equal density values could reproduce the same effect.

Remarkably, our PET-informed results showed that considering GABA-mediated scaling of regional inhibitory gain also improved the model’s ability to simulate the characteristic neural activity of DOC patients’ brains, even though these patients owe their chronic condition to severe brain injury rather than pharmacological intervention. This observation suggests a change of excitatory-inhibitory balance in favour of inhibition, not just in the generation of haemodynamic activity pertaining to propofol anaesthesia, but more broadly as a general neurobiological mechanism for the macroscale spatiotemporal activity patterns that characterise unconsciousness - whether due to anaesthesia or brain injury. Indeed, there is evidence that physiologically awake but unconscious DOC patients show cortical OFF-periods analogous to those observed in healthy individuals during sleep^106^, possibly arising from reduced cortico-cortical connectivity and a resulting shift in excitatory-inhibitory balance towards excessive inhibition^107^, as is observed at a local level after stroke^108^. And indeed, both disorders of consciousness and general anaesthesia are known to correspond to reduced cerebral metabolism, as measured with PET^109,110^.

Nevertheless, a key difference emerged between anaesthesia and DOC: whereas anaesthesia critically depends on propofol’s specific pattern of local inhibition across the cortex, incorporating regional specificity of GABA receptor density distribution did not further improve the model’s ability to simulate DOC patients’ functional connectivity dynamics, beyond the improvement provided by using a uniform or scrambled GABA-A receptor map. Therefore, whereas our results suggest that propofol anaesthesia may be causally mediated by GABA-A receptors and their specific distribution across the cortex, it appears that a global increase in inhibition is sufficient to generate the characteristic neural activity of disorders of consciousness.

Our results from connectome replacement point to injury-induced randomisation of the connectome as one such candidate mechanism in DOC patients. Specifically, our findings show that (a) unconscious fMRI functional connectivity dynamics (whether due to propofol anaesthesia or brain injury) are more compatible with the empirical DOC connectome, than conscious functional connectivity dynamics; and (b) unconscious functional connectivity dynamics are also more compatible with a random connectome than conscious ones, whereas the opposite holds for a lattice-like connectome (i.e., a regular network, the topological opposite of a random network), at least in the case of anaesthesia.

It is also remarkable that the same results from connectome replacement - greater compatibility of unconscious neural activity with the DOC connectome perturbation - could be generalised to the propofol dataset. For DOC patients, such a result may perhaps be expected, since the initial connectome used in the model was obtained from healthy controls, whereas the perturbed DOC connectome was obtained by combining the individual connectomes of the same DOC patients. However, observing the same result in the propofol dataset is a powerful validation of our approach, demonstrating that the results are specific to the presence vs absence of consciousness, rather than being influenced by the specific dataset used. Thus, thanks to connectome replacement we can infer that the increased neuronal inhibition that characterises both disorders of consciousness and anaesthesia, is functionally equivalent to randomisation of the connectome. However, propofol’s anaesthetic effects are mediated by GABA-A receptors according to their specific regional distribution, whereas disorders of consciousness can be explained in terms of a more generic increase in global inhibition - possibly arising from randomisation of the connectome due to anatomical lesions, whose extent and location are do not follow uniform patterns. Anaesthesia may be expected to operate similarly across individuals, in terms of which regions are more or less affected by propofol. In contrast, each DOC patient is unique in the cause, extent and location of their brain injury. As a result, whereas anaesthesia may depend on specific localised patterns, it stands to reason that the characteristic macroscale dynamics of DOC patients’ brains should arise from global-scale neurobiological mechanisms, which may originate from a variety of causes without necessarily depending on specific locations for injury.

Our focus here was on modelling signatures of unconsciousness that are shared across anaesthesia and disorders of consciousness; while this approach has enabled us to provide valuable insights about the neural mechanisms supporting human consciousness, this focus on common aspects means that we considered the cohort of DOC patients as a whole, both in terms of fitting the functional data and for obtaining a consensus connectome. In theory, it is possible that each patient may only exhibit increased inhibition in a specific region, but if such regions differ across patients, then considering them together may result in apparently uniform inhibition. Likewise, the similar effects of perturbation using a random connectome or the DOC connectome may in fact arise because we obtained a single DOC connectome from the combination of several patients, whose individual lesions may be specific but distinct. It is clear that the diversity of disorders of consciousness in terms of aetiology and severity can benefit from an individual-subject approach, to obtain complementary insights about each unique patient for the purposes of diagnosis, prognosis, and ultimately treatment^111^. In this regard, it is intriguing that some DOC patients can be paradoxically awakened by administration of the drug zolpidem, which is a GABA-ergic agonist^112^, which suggests that - at least for some patients - the causative neurobiological mechanisms may be substantially different from those identified here based on a group-average DOC connectome. Thus, having demonstrated the efficacy of our modelling approach at the group level, in future efforts we will build on the present results and apply the frameworks developed here to individual patients, to explore their specific deficits and potential avenues to promote recovery at a finer-grained level. Likewise, our framework could be adapted to model individual susceptibility to anaesthesia with GABA-ergic agents – and potentially predict individual risk of experiencing post-anaesthetic complications, such as emergence delirium^113,114^.

A well-known adage asserts that “All models are wrong”, and the present work is no exception. Models of neurobiological function can vary in complexity related to the level of physiological detail and scale, with both aspects incurring costs in terms of computational resources and time. Thus, trade-offs between realism and complexity are unavoidable^23^. Indeed, a variety of other modelling approaches are possible; even among DMF models, alternatives have been developed that incorporate additional information about regional neurobiology^101,115^ or use different fitting procedures^90^. More broadly, no single model can presently reproduce all relevant features of brain activity at once – in part because there is no consensus on what features of brain activity should be considered as relevant, or what the most appropriate scale for modelling is. In turn, these experimental and methodological considerations jointly shape what counts as a satisfactory model (i.e., the fitting criterion) – although here we sought to alleviate this concern by replicating our results with multiple fitting criteria. Likewise, alternative models (e.g. Hopf, Ising) have recently been used to investigate loss of consciousness during sleep^79–84^ anaesthesia^81–83,85,87,88^ and also disorders of consciousness^83,85,86^^.^ Though less neurobiologically detailed, such models have been able to provide insights about different aspects of brain function, such as criticality and the predicted effects of applying external perturbations to individual regions. Thus, it is clear that further complementary insights may be obtained by considering additional neurobiological mechanisms and multiple levels of explanation - each of which may require a different modelling approach^23,63,66^^.^

Our results combining functional MRI (dynamic macroscale neural activity), diffusion MRI (anatomical connectivity) and PET (neurotransmitter system) demonstrate that human consciousness arises from the delicate balance of local excitation and inhibition, interacting across an intricate network of anatomical connections. Many paths can lead to unconsciousness by disturbing this balance, whether by influencing the nodes’ activity (through inhibitory modulation) or the connectivity between them (through connectome randomisation). As befits such a complex dynamical system as the human brain, it is likely that other paths to unconsciousness will also exist, explaining phenomena such as regular sleep-wake alternation, epileptic seizures, and the effects of non-GABAergic anaesthetics such as ketamine – some of which have already started to be explored using whole-brain computational modelling^71,79,81,82,84^^.^ Extending the present framework to account for additional ways of losing consciousness will be a crucial endeavour. Likewise, molecular mechanisms beyond GABA-ergic inhibition provide a rich neuromodulatory landscape to support consciousness, with recent evidence indicating a common deficit of dopaminergic innervation across anaesthesia and disorders of consciousness^116^^.^ Finally, it is vital to combine multimodal neuroimaging and whole-brain modelling to identify paths from unconsciousness back to consciousness, using our understanding of post-anaesthetic recovery to restore consciousness in DOC patients, whether by means of custom-designed drugs or deep brain stimulation^66,67,89^^.^

Overall, the present findings begin to unravel the neurobiological mechanisms by which different perturbations of the brain’s structure and function - transient pharmacological intervention and chronic neuroanatomical injury - can lead to unconsciousness. Having demonstrated the power of whole-brain computational modelling to address this challenge, the same framework may also prove fruitful to address the reverse problem: namely, how the recovery of consciousness after anaesthesia can inform our ability to restore consciousness in DOC patients.

## Methods

### Anaesthesia data: Recruitment

The propofol data employed in this study have been published before^18,36,117^. For clarity and consistency of reporting, where applicable we use the same wording as our previous studies. The propofol data were collected between May and November 2014 at the Robarts Research Institute in London, Ontario (Canada)^18^. The study received ethical approval from the Health Sciences Research Ethics Board and Psychology Research Ethics Board of Western University (Ontario, Canada). Healthy volunteers (*n* = 19) were recruited (18–40 years; 13 males). Volunteers were right-handed, native English speakers, and had no history of neurological disorders. In accordance with relevant ethical guidelines, each volunteer provided written informed consent, and received monetary compensation for their time. Due to equipment malfunction or physiological impediments to anaesthesia in the scanner, data from *n* = 3 participants (1 male) were excluded from analyses, leaving a total *n* = 16 for analysis^18^.

### Anaesthesia data: Procedure

Resting-state fMRI data were acquired at different propofol levels: no sedation (Awake), and Deep anaesthesia (corresponding to Ramsay score of 5). As previously reported^18^, for each condition fMRI acquisition began after two anaesthesiologists and one anaesthesia nurse independently assessed Ramsay level in the scanning room. The anaesthesiologists and the anaesthesia nurse could not be blinded to experimental condition, since part of their role involved determining the participants’ level of anaesthesia. Note that the Ramsay score is designed for critical care patients, and therefore participants did not receive a score during the Awake condition before propofol administration: rather, they were required to be fully awake, alert and communicating appropriately. To provide a further, independent evaluation of participants’ level of responsiveness, they were asked to perform two tasks: a test of verbal memory recall, and a computer-based auditory target-detection task. Wakefulness was also monitored using an infrared camera placed inside the scanner.

Propofol (a potent agonist of inhibitory GABA-A receptors^98,99^) was administered intravenously using an AS50 auto syringe infusion pump (Baxter Healthcare, Singapore); an effect-site/plasma steering algorithm combined with the computer-controlled infusion pump was used to achieve step-wise sedation increments, followed by manual adjustments as required to reach the desired target concentrations of propofol according to the TIVA Trainer (European Society for Intravenous Aneaesthesia, eurosiva.eu) pharmacokinetic simulation program. This software also specified the blood concentrations of propofol, following the Marsh 3-compartment model, which were used as targets for the pharmacokinetic model providing target-controlled infusion. After an initial propofol target effect-site concentration of 0.6 µg mL^−1^, concentration was gradually increased by increments of 0.3 µg mL^1^, and Ramsay score was assessed after each increment: a further increment occurred if the Ramsay score was lower than 5. The mean estimated effect-site and plasma propofol concentrations were kept stable by the pharmacokinetic model delivered via the TIVA Trainer infusion pump. Ramsay level 5 was achieved when participants stopped responding to verbal commands, were unable to engage in conversation, and were rousable only to physical stimulation. Once both anaesthesiologists and the anaesthesia nurse all agreed that Ramsay sedation level 5 had been reached, and participants stopped responding to both tasks, data acquisition was initiated. The mean estimated effect-site propofol concentration was 2.48 (1.82–3.14) µg mL^−1^, and the mean estimated plasma propofol concentration was 2.68 (1.92–3.44) µg mL^−1^. Mean total mass of propofol administered was 486.58 (373.30–599.86) mg. These values of variability are typical for the pharmacokinetics and pharmacodynamics of propofol. Oxygen was titrated to maintain SpO_2_ above 96%.

At Ramsay 5 level, participants remained capable of spontaneous cardiovascular function and ventilation. However, the sedation procedure did not take place in a hospital setting; therefore, intubation during scanning could not be used to ensure airway security during scanning. Consequently, although two anaesthesiologists closely monitored each participant, scanner time was minimised to ensure return to normal breathing following deep sedation. No state changes or movement were noted during the deep sedation scanning for any of the participants included in the study^18^.

### Anaesthesia data: Design

As previously reported^18^, once in the scanner participants were instructed to relax with closed eyes, without falling asleep. Resting-state functional MRI in the absence of any tasks was acquired for 8 min for each participant. A further scan was also acquired during auditory presentation of a plot-driven story through headphones (5 min long). Participants were instructed to listen while keeping their eyes closed. The present analysis focuses on the resting-state data only; the story scan data have been published separately^87^ and will not be discussed further here.

### Anaesthesia data: FMRI data acquisition

As previously reported^18^, MRI scanning was performed using a 3-Tesla Siemens Tim Trio scanner (32-channel coil), and 256 functional volumes (echo-planar images, EPI) were collected from each participant, with the following parameters: slices = 33, with 25% inter-slice gap; resolution = 3 mm isotropic; TR = 2000 ms; TE = 30 ms; flip angle = 75 degrees; matrix size = 64 × 64. The order of acquisition was interleaved, bottom-up. Anatomical scanning was also performed, acquiring a high-resolution T1- weighted volume (32-channel coil, 1 mm isotropic voxel size) with a 3D MPRAGE sequence, using the following parameters: TA = 5 min, TE = 4.25 ms, 240 × 256 matrix size, 9 degrees flip angle^18^.

### Disorders of consciousness patient data: Overview

The DOC patient data employed in this study have been published before^18,29,58,118^. For clarity and consistency of reporting, where applicable we use the same wording as our previous studies.

### Disorders of consciousness patient data: Recruitment

A total of 71 DOC patients were recruited from specialised long-term care centres from January 2010 to December 2015^18^. Ethical approval for this study was provided by the National Research Ethics Service (National Health Service, UK; LREC reference^99^/391). Patients were eligible to be recruited in the study if they had a diagnosis of chronic disorder of consciousness, provided that written informed consent to participation was provided by their legal representative, and provided that the patients could be transported to Addenbrooke’s Hospital (Cambridge, UK). The exclusion criteria included any medical condition that made it unsafe for the patient to participate, according to clinical personnel blinded to the specific aims of the study; or any reason that made a patient unsuitable to enter the MRI scanner environment (e.g., non-MRI-safe implants). Patients were also excluded based on substantial pre-existing mental health problems, or insufficient fluency in the English language prior to their injury. After admission to Addenbrooke’s Hospital, each patient underwent clinical and neuroimaging testing, spending a total of five days in the hospital (including arrival and departure days). Neuroimaging scanning took place at the Wolfson Brain Imaging Centre (Addenbrooke’s Hospital, Cambridge, UK), and medication prescribed to each patient was maintained during scanning.

For each day of admission, Coma Recovery Scale-Revised (CRS-R) assessments were recorded at least daily. Patients whose behavioural responses were not indicative of awareness at any time, were classified as UWS. In contrast, patients were classified as being in a minimally conscious state (MCS) if they provided behavioural evidence of simple automatic motor reactions (e.g., scratching, pulling the bed sheet), visual fixation and pursuit, or localisation to noxious stimulation. Since this study focused on whole-brain properties, coverage of most of the brain was required, and we followed the same criteria as in our previous studies:^18,29^ before analysis took place, patients were systematically excluded if an expert neuroanatomist blinded to diagnosis judged that they displayed excessive focal brain damage (over one third of one hemisphere), or if brain damage led to suboptimal segmentation and normalisation, or due to excessive head motion in the MRI scanner (exceeding 3 mm translation or 3 degrees rotation). One additional patient was excluded due to incomplete acquisition. Out of the initial sample of 71 patients who had been recruited, a total of *n* = 21 adults (13 males; 17–70 years; mean time post injury: 13 months) meeting diagnostic criteria for unresponsive wakefulness syndrome/vegetative state (UWS; *N* = 10) or minimally conscious state (MCS; *N* = 11) due to brain injury were included in this study (Table 1). In addition to the researcher and radiographer, a research nurse was also present during scanning. Since the patients’ status as DOC patients was evident, no researcher blinding was possible.

**Table 1:** Demographic information for patients with disorders of consciousness.

Sex	Age	Aetiology	Diagnosis	CRS-R Score	Scan
M	46	TBI	UWS	6	12 dir
M	57	TBI	MCS	12	12 dir
M	35	Anoxic	UWS	8	12 dir
M	17	Anoxic	UWS	8	12 dir
F	31	Anoxic	MCS	10	12 dir
F	38	TBI	MCS	11	12 dir
M	29	TBI	MCS	10	63 dir
M	23	TBI	MCS	7	63 dir
F	70	Cerebral bleed	MCS	9	63 dir
F	30	Anoxic	MCS	9	63 dir
F	36	Anoxic	UWS	8	63 dir
M	22	Anoxic	UWS	7	63 dir
M	40	Anoxic	UWS	7	63 dir
F	62	Anoxic	UWS	7	63 dir
M	46	Anoxic	UWS	5	63 dir
M	21	TBI	MCS	11	63 dir
M	67	TBI	MCS	11	63 dir
F	55	Hypoxia	UWS	7	63 dir
M	28	TBI	MCS	8	63 dir
M	22	TBI	MCS	10	63 dir
F	28	ADEM	UWS	6	63 dir

*CRS-R* Coma Recovery Scale-Revised, *UWS* Unresponsive Wakefulness Syndrome, *MCS* Minimally Conscious State, *TBI* Traumatic Brain Injury.

### Disorders of consciousness patient data: FMRI data acquisition

As previously reported^18^, resting-state fMRI was acquired for 10 min (300 volumes, TR = 2000 ms) using a Siemens Trio 3 T scanner (Erlangen, Germany). Functional images (32 slices) were acquired using an echo planar sequence, with the following parameters: 3 × 3 × 3.75 mm resolution, TR = 2000 ms, TE = 30 ms, 78 degrees FA. Anatomical scanning was also performed, acquiring high-resolution T1-weighted images with an MPRAGE sequence, using the following parameters: TR = 2300 ms, TE = 2.47 ms, 150 slices, resolution 1 × 1 ×  1 mm.

### Disorders of consciousness patient data: Acquisition of diffusion-weighted imaging data

As we previously reported^58^, the DOC patients’ data were acquired over the course of several years, and as a result two different diffusion-weighted image acquisition schemes were used. For the first acquisition scheme, we collected 5 sets of 12 non-collinear diffusion-sensitising gradient directions, each set using a different b-value (5 b-values in total) ranging from 340 to 1590 s/mm^2^; therefore, a total of 60 diffusion-weighted volumes were acquired for each patient with this acquisition scheme. An echo planar sequence was used (TR = 8300 ms, TE = 98 ms, matrix size = 96 × 96, 63 slices, slice thickness = 2 mm, no gap, flip angle = 90°). This acquisition scheme was used for the first *n* = 6 patients (Table 1). The second, more recent acquisition scheme included 63 directions with a b-value of 1000 s/mm^2^; this acquisition scheme was adopted for all remaining DOC patients and also for all healthy controls. Each of these DWI acquisition types has been used before with DOC patients ^49,51,58^.

### Healthy control data: Overview and recruitment

We also acquired fMRI and DWI data from *n* = 20 healthy volunteers (13 males; 19–57 years), with no history of psychiatric or neurological disorders^58^. The Cambridgeshire 2 Research

Ethics Committee approved the study (LREC 08/H0308/246), and data were collected between October 2009 and September 2010. The mean age was not significantly different between healthy controls (*M* = 35.75; SD = 11.42) and DOC patients (*M* = 38.24; SD = 15.96) (*t*(39) = −0.57, *p* = 0.571, Hedges’s *g* = −0.18; permutation-based t-test).

### Healthy control data: FMRI data acquisition

Resting-state fMRI was acquired for 5:20 min (160 volumes, TR = 2000 ms) using a Siemens Trio 3 T scanner (Erlangen, Germany). The acquisition parameters were the same as those for the DOC patients: Functional images (32 slices) were acquired using an echo planar sequence, with the following parameters: 3 × 3 × 3.75 mm resolution, TR = 2000 ms, TE = 30 ms, 78 degrees FA. High-resolution T1-weighted anatomical images were also acquired, using an MPRAGE sequence with the following parameters: TR = 2300 ms, TE = 2.47 ms, 150 slices, resolution 1 × 1 × 1 mm.

### Healthy control data: Acquisition of diffusion-weighted imaging data

The diffusion-weighted acquisition scheme was the same 63-directions scheme used for the DOC patients, as described above and in previous work:^58^ TR = 8300 ms, TE = 98 ms, matrix size = 96 × 96, 63 slices, slice thickness = 2 mm, no gap, flip angle = 90°, 63 directions with a b-value of 1000 s/mm^2^.

A summary of the data included in our modelling pipelines is provided in Table 2.

**Table 2:** Summary of subjects included in modelling pipelines.

Group	*N*	M/F	Site	Data type	# fMRI volumes used	DWI directions
Awake/Propofol	16	12/4	London, ON	fMRI	250 (Awake) 250 (Propofol)	Not acquired
Healthy controls	20	13/7	Cambridge, UK	fMRI, DWI	155	63 directions, *B* = 1000
DOC patients	21	13/8	Cambridge, UK	fMRI, DWI	155	63 directions, *B* = 1000 (*n* = 15); 5 × 12 directions, B ranging from 340 to 1590 (*n* = 6)

Awake and propofol data were acquired from the same subjects, respectively before and after infusion with the anaesthetic. For the DOC patients, 300 volumes were acquired in total, but to ensure consistency with controls we only used 155 consecutive volumes from each patient. Note that no direct comparisons were performed between data acquired in London, ON and data acquired in Cambridge, UK.

### Functional MRI preprocessing and denoising

We followed the preprocessing pipeline described in our previous work^18^, which is based on the standard pipeline implemented within the SPM12-based (http://www.fil.ion.ucl.ac.uk/spm) CONN toolbox (http://www.nitrc.org/projects/conn), version 17 f^119^. The following steps are included in the standard CONN pipeline: removal of the first five scans to allow magnetisation to reach steady state; functional realignment and motion correction; slice-timing correction to account for differences in time of acquisition between slices; identification of outlier scans for subsequent regression by means of the quality assurance/artifact rejection software *Artifact Detection Toolbox (art;* (http://www.nitrc.org/projects/artifact_detect); spatial normalisation to MNI-152 standard space with 2 mm isotropic resampling resolution, using each volunteer’s high-resolution T1-weighted image to obtain each individual’s segmented grey matter, combined with an a priori grey matter template.

Given the presence of brain injury and corresponding deformations, DOC patients’ brains were individually preprocessed using SPM12, with visual inspections after each step. Additionally, to further reduce potential movement artifacts, data underwent despiking using the hyperbolic tangent squashing function from the CONN toolbox^119^. This method applies a continuous squashing function to the BOLD signal, rather than utilizing an absolute threshold that would result in cropping any values above that threshold. Since the controls had a shorter scan duration than DOC patients, the number of DOC functional volumes were truncated to be the same as the control subjects’ ones, after removal of the initial volumes to achieve steady-state magnetisation of the scanner, in order to ensure comparability between the two sets of Cambridge-acquired data (note that we do not perform direct comparisons between the Cambridge data and the data from London, Ontario).

Denoising also followed the same procedure as in our previous work:^18^ to reduce noise due to cardiac and motion artifacts we applied the anatomical CompCor method^120^ (also implemented within the CONN toolbox), by regressing out of the functional data the first five principal components attributable to each individual’s white matter signal; the first five components attributable to individual cerebrospinal fluid (CSF) signal; six subject-specific realignment parameters (three translations and three rotations) as well as their first-order temporal derivatives; the artifacts identified by *art;* and main effect of scanning condition^120^. Finally, after linear detrending, each individual’s denoised BOLD signal timeseries were band-pass filtered in the 0.008−0.09 Hz.range, to eliminate both low-frequency drift effects and high-frequency noise.

### DWI preprocessing and tractography

The diffusion data were preprocessed with MRtrix3 tools, using the following steps (this is the same pipeline adopted in our previous work;^58,121^ for clarity and consistency of reporting, where applicable we use the same wording as in our previous publications). After manually removing diffusion-weighted volumes with substantial distortion^51^, the pipeline involved the following steps: (i) DWI data denoising by exploiting data redundancy in the PCA domain^122^ (*dwidenoise* command); (ii) Correction for distortions induced by eddy currents and subject motion by registering all DWIs to b0, using FSL’s *eddy* tool (through MRtrix3 *dwipreproc* command); (iii) rotation of the diffusion gradient vectors to account for subject motion estimated by *eddy;*^123^ (iv) b1 field inhomogeneity correction for DWI volumes (*dwibiascorrect* command); (v) generation of a brain mask through a combination of MRtrix3 *dwi2mask* and FSL *BET* commands.

After preprocessing, the DTI data were reconstructed using the model-free q-space diffeomorphic reconstruction algorithm (QSDR) implemented in DSI Studio (www.dsi-studio.labsolver.org)^124^, following our previous work^58,125^. Use of QSDR is desirable when investigating group differences^52,124,126^ because this algorithm preserves the continuity of fiber geometry for subsequent tracking^124^, since it reconstructs the distribution of the density of diffusing water in standard space. This approach has therefore been adopted in previous connectomics studies focusing on healthy individuals^127^ but also brain-injured patients^128^ and DOC patients specifically^52,58^. QSDR initially reconstructs DWI data in native space, and subsequently computes values of quantitative anisotropy (QA) in each voxel, based on which DSI Studio performs a nonlinear warp from native space to a template QA volume in Montreal Neurological Institute (MNI) space. Once in MNI standard space, spin density functions are reconstructed, with a mean diffusion distance of 1.25 mm with three fiber orientations per voxel^124^.

Finally, fiber tracking was carried out by means of DSI Studio’s own FACT deterministic tractography algorithm, requesting 1000,000 streamlines according to widely adopted parameters:^58,125,127–129^ angular cutoff = 55^◦^, step size = 1.0 mm, tract length between 10 mm (minimum) and 400 mm (maximum), no spin density function smoothing, and QA threshold determined by DWI signal in the cerebro-spinal fluid. Streamlines were automatically rejected if they presented improper termination locations, based on a white matter mask automatically generated by applying a default anisotropy threshold of 0.6 Otsu’s threshold to the anisotropy values of the spin density function^125,127,129^.

### Brain parcellation

For both BOLD and DWI data, brains were parcellated into 68 cortical regions of interest (ROIs), according to the Desikan-Killiany anatomical atlas^130^, in line with previous whole-brain modelling work^101^.

### Functional connectivity dynamics

Following Deco et al. (2018)^89^, functional connectivity dynamics (FCD) were quantified in terms of Pearson correlation between regional BOLD timeseries, computed within a sliding window of 30 TRs with increments of 3 TRs. Subsequently, the resulting matrices of functional connectivity at times t_x_ and t_y_ were themselves correlated, for each pair of timepoints t_x_ and t_y_, thereby obtaining an FCD matrix of time-versus-time correlations. Thus, each entry in the FCD matrix represents the similarity between functional connectivity patterns at different points in time.

### Consensus group structural connectivity

The structural connectivity (SC) for the DMF model was obtained by following the procedure described in Wang et al. (2019)^101^ to derive a group-consensus structural connectivity matrix. Separately for the healthy controls and DOC patients, a consensus matrix *C* was obtained as follows. For each pair of regions *i* and *j*, if more than half of subjects had non-zero connection *i* and *j*, *C*_*ij*_ was set to the average across all subjects with non-zero connections between *i* and *j*. Otherwise, *C*_*ij*_ was set to zero. Note that this procedure for constructing a group-consensus connectome will retain or exclude a given connection between two regions only if it is present (respectively, absent) in the majority of individuals in the cohort, such that the final consensus connectome is not expected to reflect the idiosyncrasies of individual patients, but only systematic patterns.

### Whole-brain computational modelling

Whole-brain spontaneous brain activity (as quantified using blood oxygen level dependent (BOLD) signal data from functional MRI) was simulated using a neurobiologically realistic Dynamic Mean Field (DMF) model^131^. The DMF model^19,22,25^ uses an empirically validated mathematical mean-field approach to represent the collective behaviour of integrate-and-fire neurons by means of coupled differential equations, providing a neurobiologically plausible account of regional neuronal firing rate.

Specifically, the model simulates local biophysical dynamics of excitatory (NMDA) and inhibitory (GABA) neuronal populations, interacting over long-range neuroanatomical connections (white matter tracts obtained from diffusion MRI). The model further incorporates multimodal neuroimaging information about empirical brain dynamics (measured using functional MRI) and neurotransmitter receptor density, estimated from positron emission tomography (PET)^89^.

Each cortical area *n* (defined by a parcellation scheme) is represented in terms of two reciprocally coupled neuronal masses, one excitatory and the other inhibitory, with the synaptic connections between excitatory neuronal populations in different regions given by the weight of structural connectivity, to account for the number and density of interregional axon fibers. The DMF model has only one free parameter: a global coupling parameter, denoted by *G*, which scales the excitatory-to-excitatory coupling between brain regions, as established by the empirical structural connectome. Additional factors that can influence the long-range excitatory-to-excitatory coupling between brain regions, such as neurotransmission but also synaptic plasticity mechanisms, are accounted for by this global coupling parameter. Since conductivity of the white matter fibers is assumed to be constant across the brain, *G* constitutes the only free parameter in the model.

The following differential equations therefore govern the model’s behaviour:

1 $${I}_{n}^{(E)}\,=\,{W}_{E}{I}_{0}\,+\,{w}_{+}{J}_{{NMDA}}{S}_{n}^{(E)}+{{GJ}}_{{NMDA}}\mathop{\sum}\limits_{p}^{N}{C}_{{np}}{S}_{p}^{(E)}\,-\,{J}_{n}^{{FIC}}{S}_{n}^{(I)}$$

2 $${I}_{n}^{(I)}\,=\,{W}_{I}{I}_{0}\,+\,{J}_{{NMDA}}{S}_{n}^{(E)}-{S}_{n}^{(I)}$$

3 $${r}_{n}^{(E)}\,=\,F\left({I}_{n}^{(E)}\right)\,=\,\frac{{g}_{E}\left({I}_{n}^{(E)}-\,{I}_{{thr}}^{(E)}\right)}{1\,-\,{\exp }\,\left(-{d}_{E}{g}_{E}\left({I}_{n}^{(E)}-\,{I}_{{thr}}^{(E)}\right)\right)}$$

4 $${r}_{n}^{(I)}\,=\,F\left({I}_{n}^{(I)}\right)\,=\,\frac{{{g}_{n}^{{NM}}g}_{I}\left({I}_{n}^{(I)}-\,{I}_{{thr}}^{(I)}\right)}{1\,-\,{\exp }\,\left(-{d}_{I}{{g}_{n}^{{NM}}g}_{I}\left({I}_{n}^{(I)}-\,{I}_{{thr}}^{(I)}\right)\right)}$$

5 $$\frac{d{S}_{n}^{(E)}(t)}{{dt}}\,=\,\frac{{S}_{n}^{(E)}}{{\tau }_{{NMDA}}}\,+\,\left(1\,+\,{S}_{n}^{(E)}\right)\,\gamma {r}_{n}^{(E)}\,+\sigma {\nu }_{n}(t)$$

6 $$\frac{d{S}_{n}^{(I)}(t)}{{dt}}\,=\,\frac{{S}_{n}^{(I)}}{{\tau }_{{{GABA}}_{A}}}\,+\,{r}_{n}^{(I)}\,+\sigma {\nu }_{n}\left(t\right)$$

7 $${g}_{n}^{{NM}}\,=\,1\,+\,{s}_{I}{d}_{n}^{{GABA}}$$

Following previous work^89,91^, “for each excitatory *(E)* and inhibitory *(I)* neural mass, the quantities ${I}_{n}^{(E.I)}$, ${r}_{n}^{(E,I)}$, and ${S}_{n}^{(E,I)}$ represent its total input current (nA), firing rate (Hz) and synaptic gating variable, respectively. The function F(·) is the transfer function (or *F–I curve*), representing the non-linear relationship between the input current and the output firing rate of a neural population. Finally, ${J}_{n}^{{FIC}}$ is the local feedback inhibitory control of region *n*, which is optimized to keep its average firing rate at approximately 3 Hz^25,91^, and ${\nu }_{n}$ is uncorrelated Gaussian noise injected to region *n*”. The model’s fixed parameters are reported in Table 3^25,89,91^. Additionally, ${g}_{n}^{{NM}}$ is the neuromodulatory scaling factor modulating the transfer function for each cortical region in the model as a function of ${d}_{n}^{{GABA}}$, the regional density of GABA-A receptors (see below for details) and an inhibitory gain scaling parameter ${s}_{I}$. The original DMF model (corresponding to a DMF model with uniform GABA-A regional inhibitory gain, and no regionally heterogeneous neuromodulation) is obtained by setting ${s}_{I}$to zero, in which case *G* remains the sole free parameter in the model. Details for optimisation of the ${s}_{I}$ parameter for the GABA-A modulated model are provided below.

**Table 3:** Dynamic Mean Field model parameters.

Parameter	Symbol	Value
External current	I_0_	0.382 nA
Excitatory scaling factor for I_0_	W_E_	1
Inhibitory scaling factor for I_0_	W_I_	0.7
Local excitatory recurrence	w_+_	1.4
Excitatory synaptic coupling	J_NMDA_	0.15 nA
Threshold for F (I_n_^(E)^)	I_thr_^(E)^	0.403 nA
Threshold for F (I_n_^(I)^)	I_thr_^(I)^	0.288 nA
Gain factor of F (I_n_^(E)^)	g_E_	310 nC^−1^
Gain factor of F (I_n_^(I)^)	g_I_	615 nC^-1^
Shape of F (I_n_^(E)^) around I_thr_^(E)^	d_E_	0.16 s
Shape of F (I_n_^(I)^) around I_thr_^(I)^	d_I_	0.087 s
Excitatory kinetic parameter	$\gamma$	0.641
Amplitude of uncorrelated Gaussian noise v_n_	$\sigma$	0.01 nA
Time constant of NMDA	${\tau }_{{NMDA}}$	100 ms
Time constant of GABA	${\tau }_{{{GABA}}_{A}}$	10 ms

A Balloon-Windkessel (BW) hemodynamic model^97^ was then used to turn simulated regional neuronal activity into simulated regional BOLD signal. The Balloon-Windkessel model considers the BOLD signal as a nonlinear function of the normalized total deoxyhemoglobin voxel content, normalized venous volume, resting net oxygen extraction fraction by the capillary bed, and resting blood volume fraction. The BOLD-signal estimation for each brain area is computed from the level of neuronal activity in that particular area. Finally, simulated regional BOLD signal was bandpass filtered in the same range as the empirical data (0.008–0.09 Hz).

### Implementation

The code used to run all the simulations in this study was written in optimised C++ using the high-performance library Eigen. The C++ core of the code, together with Python and Octave/Matlab interfaces is publicly available as “FastDMF”^131^ and maintained at http://www.gitlab.com/concog/fastdmf.

To simulate BOLD data, FastDMF splits the problem in two steps: integrating the coupled differential equations underlying the DMF model, to obtain excitatory firing rates in each brain region; and using these firing rates to integrate the (uncoupled) differential equations of the BW hemodynamic model and obtain BOLD timeseries.

Integration of the DMF equations is performed with the Euler-Maruyama method, and it is highly parallelizable and bounded by the O(N^2) complexity of the matrix-vector multiplication corresponding to the excitatory-to-excitatory coupling between brain regions. Simulated excitatory firing rates are stored in a cylindrical array with a fixed buffer size to limit memory requirements.

In addition, a further set of threads is spawned to solve the BW model using the simulated excitatory firing rates. Since the BW solver reads from the same cylindrical array, it interfaces with the DMF solver with a controlled multi-threaded architecture. Every TR-equivalent in simulation time the value of all BOLD signals is copied to a pre-allocated array, to be returned at the end of the requested simulation time.

In a standard laptop, FastDMF attains a speed-up of between 5x and 10x over publicly available Matlab implementations, due to the speed of Eigen and the parallelisation of DMF and BW solvers. In addition, due to the cylindrical buffer, this implementation is able to simulate arbitrarily long BOLD time series with a fixed memory overhead, thereby allocating orders of magnitude less memory than a naive Matlab implementation.

Finally, the library includes interface functions for Matlab (via its C Matrix API) and Python (via the Boost.Python library). In both languages the function returns a standard array (numpy.ndarray in the case of Python) that can be easily processed for further analysis.

### Fitting of the *G* parameter

Calibrating the model corresponds to finding the value of *G* that allows the model to best simulate observed fMRI activity patterns of the human brain at rest. In order to identify appropriate parameters for the simulations, early whole-brain modelling efforts used the grand average FC as target for fitting the model to empirical data. However, it has since become apparent that the macroscale neural signals measured by functional MRI are not static, even on the timescale of a few tens of seconds: rather, they exhibit a wide range of dynamic patterns. Therefore, in order to properly take into account the time-dependencies of FC, it is advantageous to fit the model to empirical functional connectivity dynamics (FCD). Doing so ensures that the simulated BOLD data will exhibit realistic patterns of time-evolving functional connectivity^89,96^.

Unlike matrices of inter-regional connectivity, where each brain region is the same across different scans, FCDs are represented as matrices encoding the relationship between brain dynamics at different timepoints. Since timepoints are not the same across individuals or scans, as our functional MRI data were acquired under conditions of task-free rest rather than being time-locked to a particular event, FCD matrices cannot be compared by means of simple correlation. Therefore, to evaluate model performance in terms of producing meaningful temporal dynamics, here we follow the approach of Deco et al. (2018)^89^, using the Kolmogorov-Smirnov distance to compare the histograms of empirical and simulated FCD values (obtained from the upper triangular FCD matrix), to find the *G* parameter that results in the best match between empirical and simulated functional connectivity dynamics. The same KS-distance was also used as the goodness-of-fit measure to quantify the similarity between empirical and simulated macroscale brain activity.

To find the value of *G* that generates simulations whose FCD best match empirical FCD, we generated n = 100 simulations for each value of *G* between 0.1 and 2.5, using increments of 0.1. For each simulation at each value of *G*, we computed the KS distance between empirical (group-wise) and simulated FCD. Finally, we set the model’s *G* parameter to the value that minimised the mean KS distance - corresponding to the model that is best capable of simulating the temporal dynamics of functional connectivity observed in the healthy human brain at rest.

This procedure was performed separately for the propofol dataset (with 250 TRs) and the DOC dataset, which was truncated to the number of TRs available for the healthy controls (155 TRs). For validation, we also replicated our main results when the traditional KS-distance was replaced with an alternative, two-dimensional version of the KS-distance as the chosen goodness-of-fit measure. This alternative measure, introduced by Peacock (1983)^132^, was used to take into account not only the distribution of inter-temporal correlation values (i.e., the values in the FCD matrix), but also their relative temporal position with respect to each other, in terms of the number of intervening sliding-windows between them. Mathematically, this corresponds to comparing the empirical and simulated distributions *p*(*r*, $\tau$) for a given FC correlation *r* across a time-lag $\tau$.

### Local inhibitory gain modulation from GABA-A maps

Since the general anaesthetic propofol is an agonist of the GABA-A receptor, we modulated local inhibitory gain based on the recent high-resolution quantitative atlas of human brain GABA-A receptors, generated on the basis of benzodiazepine receptor (BZR) density measured in vivo from [^11^C]flumazenil Positron Emission Tomography (PET) autoradiography^100^, made available by the Neurobiology Research Unit of the Copenhagen University Hospital. Briefly, a parametric map reflecting maximal binding of [^11^C]flumazenil was obtained by averaging Logan analysis estimates based on PET data obtained from n=16 (7 males) healthy volunteers (age range: 16–46 years: M = 26.6 +/− 8 years). Data were acquired with a High-Resolution Research Tomograph (CTI/Siemens). We refer the reader to the original publication for full details of the data acquisition and map generation procedure^100^. Following^133^, after parcellating the cortical map according to the Desikan-Killiany atlas used in the present study, the data were Z-scored, before normalising the values to lie between 0 and 1.

We then used the previously calibrated DMF model to generate simulations for values of *s*_*I*_ up to 1, varying in increments of 0.02. Then, for each value of *s*_*I*_, we computed the KS distance between the model’s simulated macroscale dynamics and the empirical dynamics observed in each condition (awake or propofol, control or DOC). For each condition, the optimal value of *s*_*I*_, was then identified as the value that resulted in the minimum mean KS distance between empirical and simulated dynamics (across *n* = 10 simulations for each value of *s*_*I*_).

As validation analysis, we also repeated the same procedure, optimising the inhibitory gain scaling *s*_*I*_, but with two different kinds of receptor density maps: a scrambled map, whereby the values of GABA-A receptor density obtained from PET were randomised across regions while preserving their spatial autocorrelation^134,135^; and a uniform map, whereby each region was set to the same value, corresponding to the mean of the distribution of PET-derived receptor densities.

### Connectome replacement

Connectome replacement was performed using the initial balanced DMF model (i.e., with optimised *G* parameter, but without additional inhibitory gain modulation), based on the consensus connectome from diffusion imaging of healthy controls (referred to as the healthy connectome).

Three perturbed connectomes were used. Firstly, the consensus connectome obtained from diffusion imaging of *n* = 21 DOC patients, referred to as the DOC connectome. Secondly, the original healthy connectome was randomised according to the weight-preserving procedure of^102^ to generate a random connectome that differs from the original in terms of topology, but preserves the weight distribution. Thirdly, we used the procedure described in^102^ to turn the healthy connectome into a lattice network with the same weight distribution - providing a different and opposite perturbation of the network’s topology.

For each perturbed connectome, the DMF model was used to generate *n* = 100 simulations, using the optimal global coupling *G*, but with inter-regional connectivity given by the perturbed connectome rather than the original connectome. This was repeated for each dataset (propofol and DOC) and the resulting simulations were compared with each condition (awake/propofol and control/DOC) in terms of KS-distance.

### Statistics and reproducibility

Statistical differences were evaluated for significance at the standard alpha level of 0.05 (two-sided), using permutation-based between-subjects t-tests on the distributions of KS-distance values obtained from *n* = 100 simulations from the corresponding models being compared Mean and standard error of the mean of the data are displayed in the Figures. Supplementary Tables 1–4 report the test results. Effect sizes were estimated as Cohen’s *d* (standardised difference of means).

### Reporting summary

Further information on research design is available in the Nature Research Reporting Summary linked to this article.

## Supplementary information

Supplementary Information (new)

Description of Additional Supplementary Files

Supplementary Data 1

Supplementary Data 2

Supplementary Data 3

Supplementary Data 4

Reporting Summary

## Data Availability

Source data underlying Figs. 2b and 3 are presented in Supplementary Data 1. Source data underlying Fig. 4 are presented in Supplementary Data 2. Source data underlying Fig. 5 are presented in Supplementary Data 3. Source data underlying Fig. 6 are presented in Supplementary Data 6. The propofol and DOC patient data that support the findings of this study are available from Dr. Emmanuel Stamatakis, University of Cambridge (email: eas46@cam.ac.uk) upon reasonable request. The GABA PET maps are available from the Neurobiology Research Unit at Copenhagen University Hospital (https://xtra.nru.dk/BZR-atlas/).

## Code availability

The C++ core of the DMF code, together with Python and Octave/Matlab interfaces, has been made publicly available^131^, and it is actively maintained at http://www.gitlab.com/concog/fastdmf. The CONN toolbox is freely available online (http://www.nitrc.org/projects/conn). DSI Studio is freely available online: dsi-studio.labsolver.org. MRtrix3 is freely available online: https://www.mrtrix.org. The *abagen* toolbox is freely available online: https://abagen.readthedocs.io/en/stable/.
