# Supplementary material for: Whole-brain modelling identifies distinct but convergent paths to unconsciousness in anaesthesia and disorders of consciousness
Source: Commun Biol. 2022 Apr 20;5:384. doi: 10.1038/s42003-022-03330-y (PMC9021270; doi:10.1038/s42003-022-03330-y)
Supplement: Supplementary file 1 — Supplementary Information (new) [file 42003_2022_3330_MOESM1_ESM.pdf]

Whole-Brain Modelling Identifies Distinct but Convergent Paths to Unconsciousness in Anaesthesia and Disorders of Consciousness

Supplementary Information

Andrea I. Luppi[1,2,3,4]*, Pedro A.M. Mediano[5], Fernando E. Rosas[6,7,8], Judith Allanson[2,9], John D. Pickard[2,10], Guy B. Williams[2,11], Michael M. Craig[1,2], Paola Finoia[2], Alexander R.D. Peattie[1,2], Peter Coppola[1,2], Adrian M. Owen[l2], Lorina Naci[13], David K. Menon[1,11], Daniel Bor[5], Emmanuel A. Stamatakis[1,2]

[1]Division of Anaesthesia, School of Clinical Medicine, University of Cambridge, United Kingdom

[2]Department of Clinical Neurosciences, University of Cambridge, Cambridge, United Kingdom

[3]Leverhulme Centre for the Future of Intelligence, University of Cambridge, Cambridge, United Kingdom

[4]The Alan Turing Institute, London, United Kingdom

[5]Department of Psychology, University of Cambridge, Cambridge, United Kingdom

[6]Center for Psychedelic Research, Department of Brain Science, Imperial College London, London, United Kingdom

[7]Data Science Institute, Imperial College London, London, United Kingdom

[8]Centre for Complexity Science, Imperial College London, London, United Kingdom

[9]Department of Neurosciences, Cambridge University Hospitals NHS Foundation, Addenbrooke's Hospital, Cambridge, United Kingdom

[10]Division of Neurosurgery, School of Clinical Medicine, University of Cambridge, Cambridge, United Kingdom

[11]Wolfson Brain Imaging Centre, University of Cambridge, Cambridge, United Kingdom

[12]The Brain and Mind Institute, University of Western Ontario, London, Ontario, Canada

[13]Trinity College Institute of Neuroscience, Trinity College Dublin, Dublin, Ireland

*Corresponding author; email address: al857@cam.ac.uk

Supplementary Figures

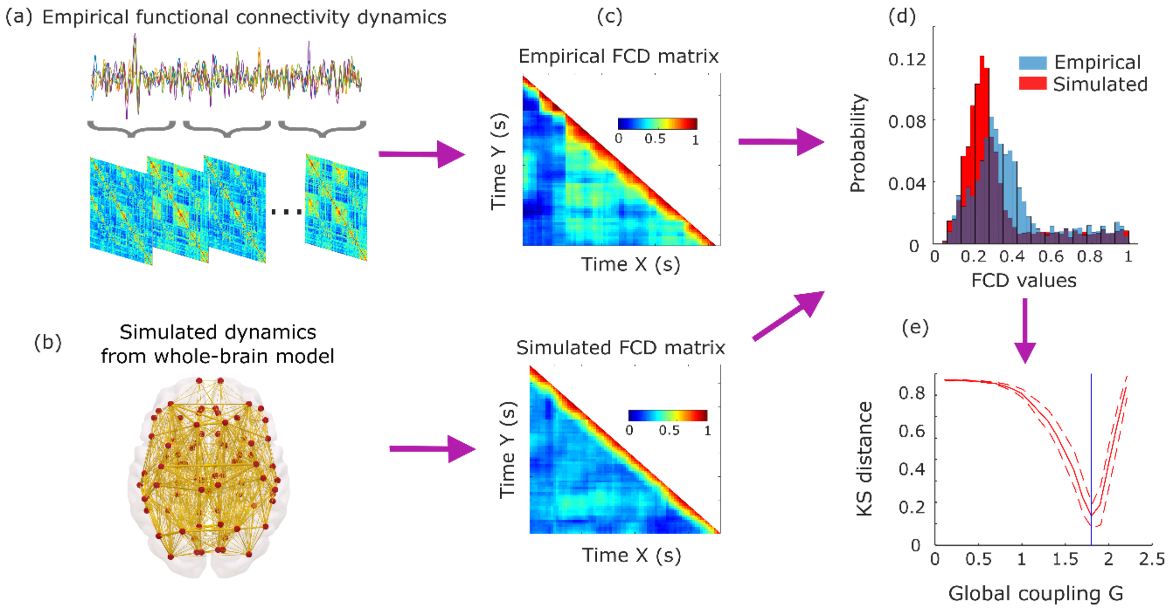

Supplementary Figure 1. Overview of model fitting procedure. (a) Time-resolved matrices of functional connectivity are obtained from empirical functional MRI via the sliding-window approach: regional BOLD time-series are partitioned into windows of 30 TRs, sliding by 3 TRs at a time, following the same approach as previous work using the DMF model; functional connectivity between each pair of regions is computed within each window by means of Pearson correlation, generating a stack of FC matrices representing the evolution of FC over time. (b) The same procedure is repeated for the simulated BOLD timeseries produced by the model with various levels of the global coupling parameter, *G*. (c) For both the empirical and simulated functional connectivity dynamics (FCD), a time-versus-time FCD matrix is computed by correlating the time-dependent FC matrices centred at each timepoint. (d) Histograms of the distribution of FCD values in each matrix are obtained over all participants (blue) and for each simulation (red), and their similarity is evaluated by means of the Kolmogorov-Smirnov distance. (e) Across values of the global coupling parameter *G*, we compute the KS-distance between the empirical FCD and the FCD of each simulation (red solid line; dashed lines indicate standard deviation). The optimum value of *G* for the model (blue vertical line) is chosen as the one that minimises the average KS-distance across n=100 simulations (here shown for the awake condition of the propofol dataset, where *G*=1.8). This procedure determines the value that allows the model to best simulate the empirical dynamics of functional connectivity in the healthy human brain.

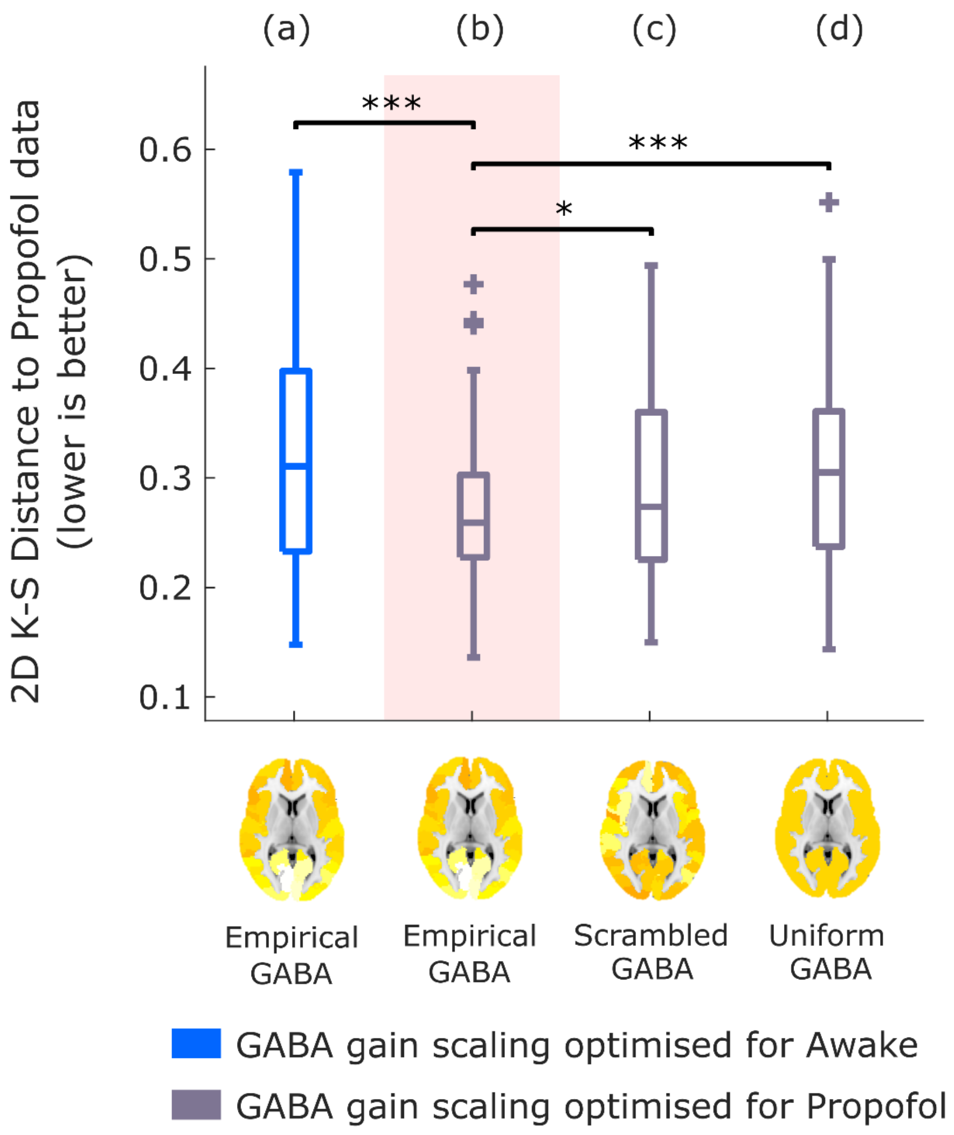

Supplementary Figure 2. Replication of results from inhibitory neuromodulation using 2-dimensional KS-distance fitting criterion. (a) The model informed by the empirical distribution of GABA-A receptors and with inhibitory gain scaling optimised to fit the Propofol data (shaded column), is compared against the same model but with inhibitory gain scaling optimised to fit the Awake data (b), or against the same model but after randomly reshuffling the regional receptor densities across the cortex (c), or setting them all to a uniform value (mean of the empirical distribution) (d). Box-plots show the model fit to the propofol data for n=100 simulations, (middle line: median; box limits, upper and lower quartiles; whiskers, 1.5x inter-quartile range; "+" symbol indicates outliers) quantified as 2D KS-distance (see Methods) between simulated and empirical FCD, for each variant of the model. * $p < 0.05$; *** $p < 0.001$.

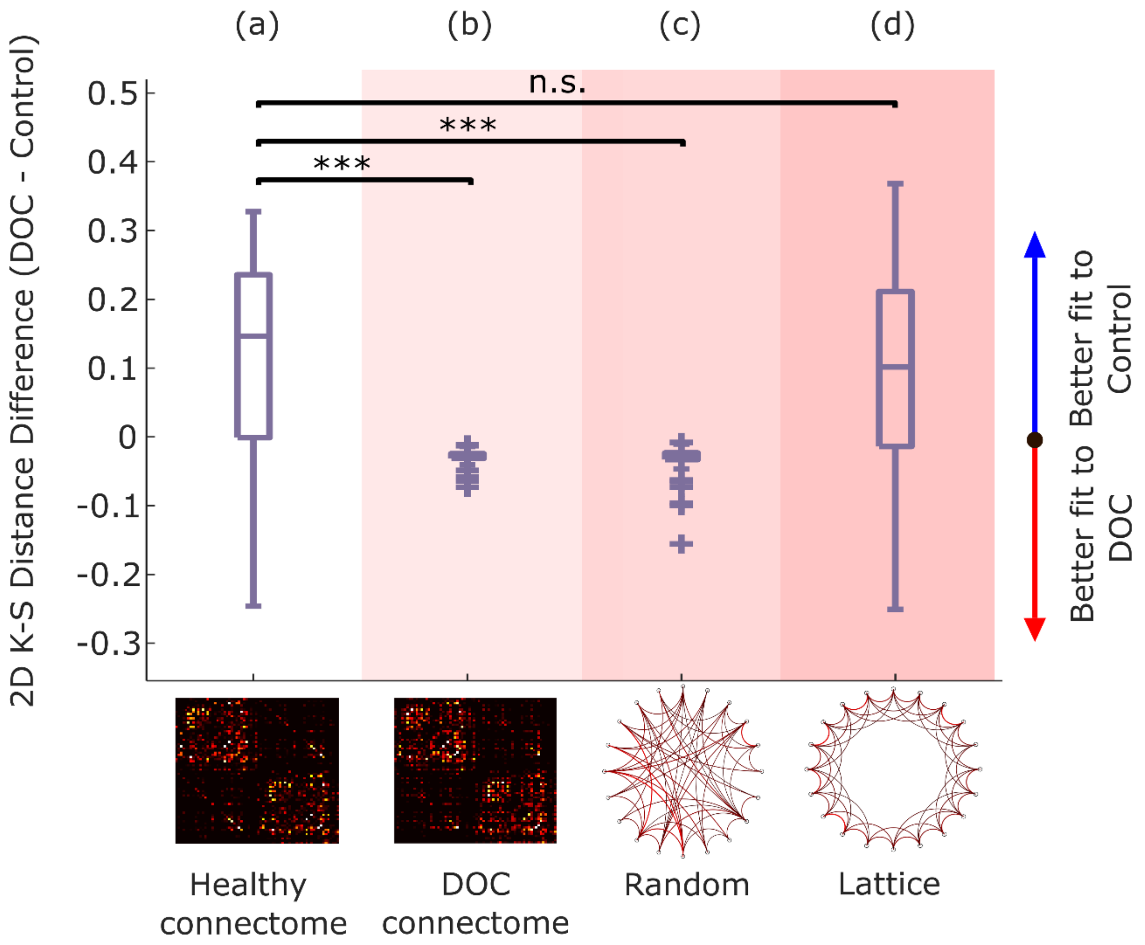

Supplementary Figure 3. Replication of connectome replacement results using 2-dimensional KS-distance fitting criterion. Box-plots show the difference in model fit (2D KS-distance; see Methods) between the two conditions (fit to DOC patients' data minus fit to healthy controls' data, over n=100 simulations), for the initial model calibrated based on the healthy connectome (a), and after replacing the model's initial connectome with either the DOC patients' empirical consensus connectome (b), or after rewiring the initial connectome into a random network (c), or into a regular (lattice) network (d). Middle line: median; box limits, upper and lower quartiles; whiskers, 1.5x inter-quartile range; "+" symbol indicates outliers; ***, $p < 0.001$; n.s., not significant ($p > 0.05$)..

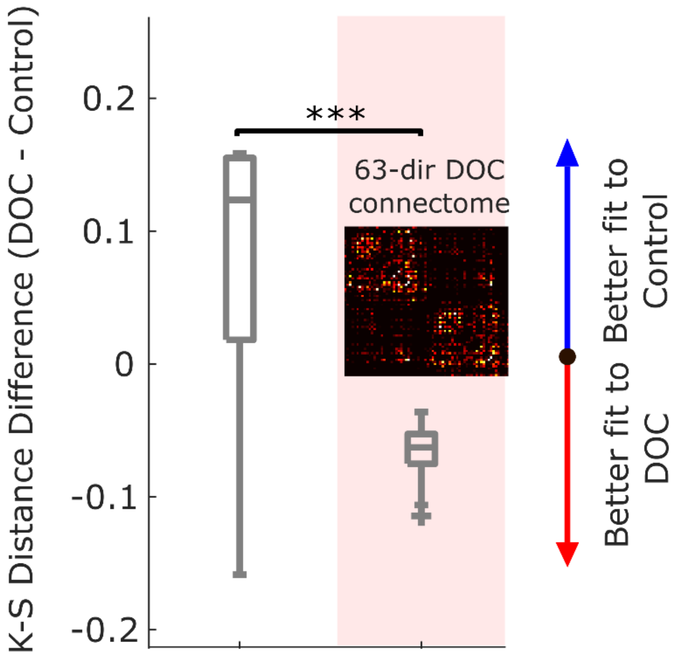

Supplementary Figure 4. Replication of connectome replacement results using a consensus DOC connectome obtained from only the patients with 63-direction diffusion MRI data. Box-plot shows the difference in model fit (KS-distance) between the two conditions (fit to DOC patients' data minus fit to healthy controls' data, over n=100 simulations), for the initial model calibrated based on the healthy connectome (left), and after replacing the model's initial connectome with either the DOC patients' empirical consensus connectome, constructed from the N=15 DOC patients with 63-direction diffusion MRI data (right). Middle line: median; box limits, upper and lower quartiles; whiskers, 1.5x inter-quartile range; "+" symbol indicates outliers; ***, $p < 0.001$

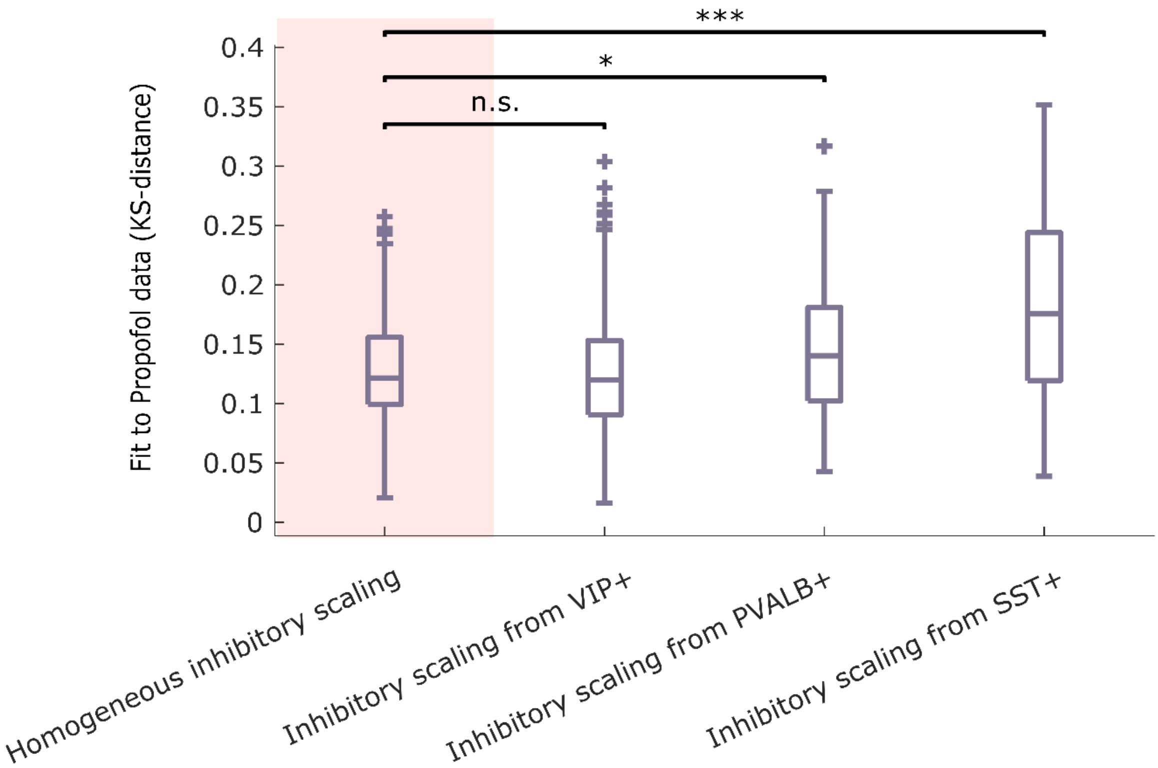

Supplementary Figure 5. Regionally heterogeneous inhibitory neuromodulation following the regional transcriptomic distribution of major inhibitory interneuron types. The model informed by the empirical distribution of GABA-A receptors and with regionally uniform inhibitory gain scaling optimised to fit the Propofol data (shaded column), is compared against the same model but with additional regionally heterogeneous inhibitory gain scaling, according to the distribution of three major types of inhibitory interneurons obtained from the Allen Institute for Brain Science transcriptomic dataset: vasoactive intestinal peptide-positive (VIP+), parvalbumin-positive (PVALB+), and somatostatin-positive (SST+). Box-plots show the model fit to the propofol data for n=100 simulations, (middle line: median; box limits, upper and lower quartiles; whiskers, 1.5x inter-quartile range; "+" symbol indicates outliers). Middle line: median; box limits, upper and lower quartiles; whiskers, 1.5x inter-quartile range; "+" symbol indicates outliers; ,* $p < 0.05$; ***, $p < 0.001$; n.s., not significant ($p > 0.05$).

Supplementary Note 1: Jacobian spectral analysis

As the dynamic mean field model we employ is nonlinear, the Jacobian is not constant but changes at every point in time. However, we adopted a recently developed method (Barter et al., 2021) to reconstruct the Jacobian of a dynamical system from time-series covariance, when there is information about the underlying network structure connecting the system's variables – which is precisely our case, since we have information about the anatomical connectivity between brain regions. The Jacobian J is related to Γ, the stationary covariance matrix of the system, and D, the covariance matrix of the fluctuations (i.e., the noise injected at each time-step), by the following relation:

$$J\Gamma+\Gamma JT=-2D \tag{1}$$

We obtained Γ numerically from simulated firing rate time-series, and D analytically from the system's parameters and differential equations. Since noise is only injected in the system via white noise of amplitude σ added to $S_n^{(E)}$and $S_n^{(I)}$, it is easy to compute D, the fluctuations covariance induced in $r_n^{(E)}$, using standard uncertainty propagation techniques. Since $I_n^{(E)}$is a linear combination of $S_n^{(E)}$, $S_n^{(I)}$, its covariance can be calculated exactly with the usual formulae for Gaussian variables. Since $r_n^{(E)}$ is a non-linear function of $I_n^{(E)}$, we perform a first-order Taylor approximation, evaluate the derivative of $r_n^{(E)}$ = F($I_n^{(E)}$) at the value of the stationary mean of $I_n^{(E)}$, and compute the induced covariance in $r_n^{(E)}$ using the same Gaussian formulae. With D and Γ in hand, we can then use the method of Barter et al (2021) and information about the connections between regions (encoded in the connectome) to reconstruct the Jacobian and subsequently obtain its eigenvalues.

To summarise, we generated time-series of regional firing rates using the models with either the original healthy connectome, the DOC patients' consensus connectome, or the rewired connectomes (random and lattice). For each of these four cases, we then used the generated regional time-series to obtain a covariance matrix, and we used this covariance matrix, together with the corresponding connectome and an estimate of the regional noise fluctuations, to reconstruct the system's Jacobian according to the method of Barter et al., (2021). We repeated this procedure n=20 times for each connectome. We observed that the Jacobian eigenvalues were complex, including both positive and negative real parts. To quantify the similarity

between these different perturbations (DOC, random and lattice connectomes) to the model's dynamics, we then measured the Spearman correlation between the real parts of the eigenvalues of the reconstructed Jacobians, resulting in an eigenvalue correlation matrix for each of the 20 repetitions . On average across repetitions, the real part of the eigenvalues of the reconstructed Jacobians from the healthy connectome shows the least similarity with the eigenvalues obtained from perturbed connectomes (DOC, random and lattice), which are more highly correlated with each other (Supplementary Figure 6).

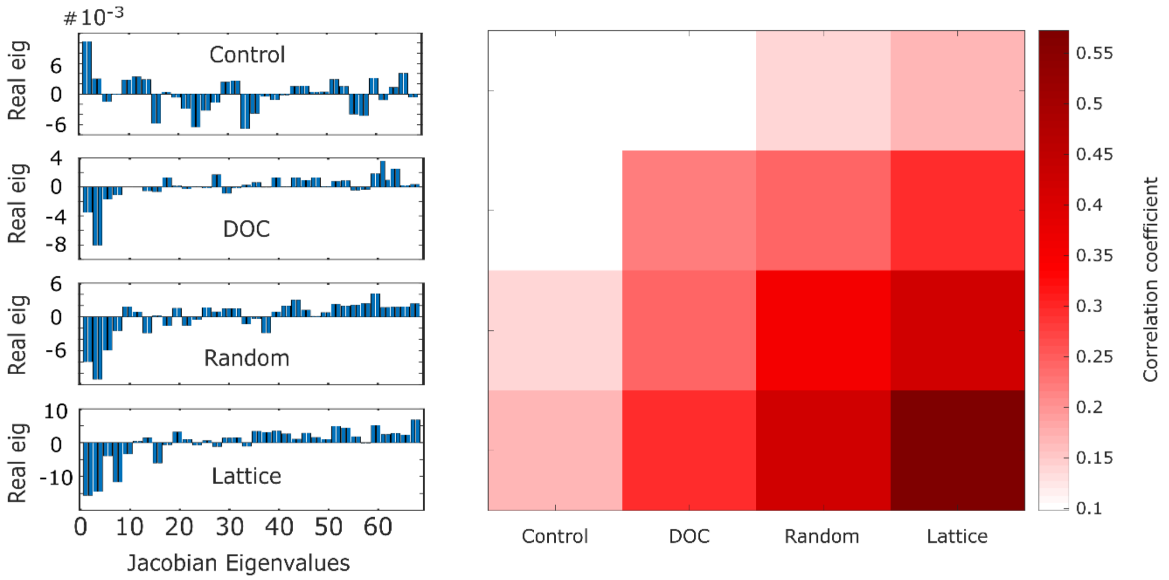

Supplementary Figure 6. Spectral analysis of reconstructed Jacobian for different connectomes. Left: example of the real part of the eigenvalues of the reconstructed Jacobian, for each connectome. Right: average correlation between the real parts of the eigenvalues from each pair of n=20 Jacobians reconstructed from each connectome.

Supplementary Tables

Supplementary Table 1. Statistical testing for the effects of local inhibitory GABA modulation, using permutation-based between-subjects t-tests. Alternative models are described in the row headers.

	**Mean (Empirical GABA)**	**Std (Empirical GABA)**	**Mean (alternative model)**	**Std (alternative model)**	**df**	**t-stat**	**p-value**	**EffSize**
**Versus Empirical GABA optimised for Awake**	0.128	0.051	0.186	0.086	198	-5.850	<0.001	-0.824
**Versus Scrambled GABA**	0.128	0.051	0.150	0.060	198	-2.842	0.005	-0.400
**Versus Uniform GABA**	0.128	0.051	0.155	0.055	198	-3.569	<0.001	-0.503

Supplementary Table 2. Statistical testing for the effects of connectome replacement, using permutation-based between-subjects t-tests. Alternative models are described in the row headers.

	**Mean (Healthy Connectome)**	**Std (Healthy Connectome)**	**Mean (alternative model)**	**Std (alternative model)**	**df**	**t-stat**	**p-value**	**EffSize**
**Versus DOC Connectome**	0.074	0.103	-0.095	0.019	198	16.107	0.000	2.269
**Versus Random Connectome**	0.074	0.103	-0.098	0.024	198	16.211	0.000	2.284
**Versus Lattice Connectome**	0.074	0.103	0.059	0.097	198	1.036	0.301	0.146

Supplementary Table 3. Generalisation of local GABA modulation results to DOC patients, using permutation-based between-subjects t-tests. Alternative models are described in the row headers.

	Mean (Empirical GABA)	Std (Empirical GABA)	Mean (alternative model)	Std (alternative model)	df	t-stat	p-value	EffSize
**Versus Empirical GABA optimised for Awake**	0.168	0.068	0.287	0.092	198	-10.43	<0.001	-1.470
**Versus Scrambled GABA**	0.168	0.068	0.158	0.066	198	1.029	0.305	0.145
**Versus Uniform GABA**	0.168	0.068	0.159	0.054	198	0.938	0.349	0.132

Supplementary Table 4. Generalisation of connectome replacement results to anaesthetised volunteers, using permutation-based between-subjects t-tests. Alternative models are described in the row headers.

	Mean (Healthy Connectome)	Std (Healthy Connectome)	Mean (alternative model)	Std (alternative model)	df	t-stat	p-value	EffSize
**Versus DOC Connectome**	0.017	0.060	-0.043	0.008	198	9.775	<0.001	1.377
**Versus Random Connectome**	0.017	0.060	-0.038	0.009	198	8.975	<0.001	1.264
**Versus Lattice Connectome**	0.017	0.060	0.049	0.042	198	-4.491	<0.001	-0.633
