# Supplementary material for: Whole-brain modelling identifies distinct but convergent paths to unconsciousness in anaesthesia and disorders of consciousness
Source: Commun Biol. 2022 Apr 20;5:384. doi: 10.1038/s42003-022-03330-y (PMC9021270; doi:10.1038/s42003-022-03330-y)
Supplement: Supplementary file 2 — Description of Additional Supplementary Files [file 42003_2022_3330_MOESM2_ESM.pdf]

Description of Additional Supplementary Files

File name: Supplementary Data 1
Description: Source data for Figures 2b and 3.

File name: Supplementary Data 2
Description: Source data for Figure 4.

File name: Supplementary Data 3
Description: Source data for Figure 5.

File name: Supplementary Data 4
Description: Source data for Figure 6.
