# Supplementary material for: Whole-brain modelling identifies distinct but convergent paths to unconsciousness in anaesthesia and disorders of consciousness
Source: Commun Biol. 2022 Apr 20;5:384. doi: 10.1038/s42003-022-03330-y (PMC9021270; doi:10.1038/s42003-022-03330-y)
Supplement: Supplementary file 7 — Reporting Summary [file 42003_2022_3330_MOESM7_ESM.pdf]

nature research

Corresponding author(s): Andrea Luppi

Last updated by author(s): March 16, 2022

Reporting Summary

Nature Research wishes to improve the reproducibility of the work that we publish. This form provides structure for consistency and transparency in reporting. For further information on Nature Research policies, see our Editorial Policies and the Editorial Policy Checklist.

Statistics

For all statistical analyses, confirm that the following items are present in the figure legend, table legend, main text, or Methods section.

n/a	Confirmed	
☐	☒	The exact sample size (n) for each experimental group/condition, given as a discrete number and unit of measurement
☒	☐	A statement on whether measurements were taken from distinct samples or whether the same sample was measured repeatedly
☐	☒	The statistical test(s) used AND whether they are one- or two-sided<br>*Only common tests should be described solely by name; describe more complex techniques in the Methods section.*
☒	☐	A description of all covariates tested
☒	☐	A description of any assumptions or corrections, such as tests of normality and adjustment for multiple comparisons
☐	☒	A full description of the statistical parameters including central tendency (e.g. means) or other basic estimates (e.g. regression coefficient) AND variation (e.g. standard deviation) or associated estimates of uncertainty (e.g. confidence intervals)
☐	☒	For null hypothesis testing, the test statistic (e.g. F, t, r) with confidence intervals, effect sizes, degrees of freedom and P value noted<br>*Give P values as exact values whenever suitable.*
☒	☐	For Bayesian analysis, information on the choice of priors and Markov chain Monte Carlo settings
☒	☐	For hierarchical and complex designs, identification of the appropriate level for tests and full reporting of outcomes
☐	☒	Estimates of effect sizes (e.g. Cohen's d, Pearson's r), indicating how they were calculated

Our web collection on statistics for biologists contains articles on many of the points above.

Software and code

Policy information about availability of computer code

Data collection: Propofol was administered using an AS50 auto syringe infusion pump (Baxter Healthcare, Singapore). The desired target concentrations of propofol according to the TIVA Trainer (European Society for Intravenous Aneaesthesia, eurosiva.eu) pharmacokinetic simulation program.

Data analysis: The C++ core of the DMF code, together with Python and Octave/Matlab interfaces, has been made publicly available at http://www.gitlab.com/concog/fastdmf.
The CONN toolbox is freely available online (http://www.nitrc.org/projects/conn).
DSI Studio is freely available online: dsi-studio.labsolver.org.
MRtrix3 is freely available online: https://www.mrtrix.org.

For manuscripts utilizing custom algorithms or software that are central to the research but not yet described in published literature, software must be made available to editors and reviewers. We strongly encourage code deposition in a community repository (e.g. GitHub). See the Nature Research guidelines for submitting code & software for further information.

Data

Policy information about availability of data

All manuscripts must include a data availability statement. This statement should provide the following information, where applicable:

- Accession codes, unique identifiers, or web links for publicly available datasets
- A list of figures that have associated raw data
- A description of any restrictions on data availability

The propofol and DOC patient data that support the findings of this study are available from Dr. Emmanuel Stamatakis, University of Cambridge (email: eas46@cam.ac.uk) upon reasonable request.
The GABA PET data are available from the Neurobiology Research Unit at Copenhagen University Hospital (https://xtra.nru.dk/BZR-atlas/).

Field-specific reporting

Please select the one below that is the best fit for your research. If you are not sure, read the appropriate sections before making your selection.

☐ Life sciences ☒ Behavioural & social sciences ☐ Ecological, evolutionary & environmental sciences

For a reference copy of the document with all sections, see nature.com/documents/nr-reporting-summary-flat.pdf

Behavioural & social sciences study design

All studies must disclose on these points even when the disclosure is negative.

Study description	This study uses quantitative methods.
Research sample	Anaesthesia dataset: 19 healthy volunteers were recruited (native English speakers, right-handed; 18–40 years; 13 males) . DOC patients: 71 patients meeting diagnostic criteria for Unresponsive Wakefulness Syndrome/Vegetative State or Minimally Conscious State were initially recruited for the study, and the final cohort analysed in this study comprised 21 individuals (13 males; 17 -70 years; mean time post injury: 13 months; 10 UWS, 11 MCS). Healthy controls (Cambridge dataset): N=20 healthy volunteers (13 males; 19-57 years), with no history of psychiatric or neurological disorders.
Sampling strategy	No power analysis was performed prior to data collection; both final sample sizes are within the range reported in the literature.
Data collection	For the DOC dataset, in addition to the researcher and radiographer, a research nurse was also present. Since the patients' status as DOC patients was evident, no researcher blinding was possible.<br>For the propofol dataset, two senior anaesthesiologists and one anaesthesia nurse were also present, and they could not be blinded to experimental condition, since part of their role involved determining the participants' level of anaesthesia.
Timing	DOC patient data were collected between January 2010 and December 2015; healthy controls for the Cambridge dataset were collected between October 2009 and September 2010; propofol data were collected between May and November 2014.
Data exclusions	Due to equipment malfunction or physiological impediments to anaesthesia in the scanner, data from three healthy volunteers (1 male) from the propofol dataset were excluded from analyses, leaving 16 volunteers for analysis.<br>Out of 71 DOC patients recruited for the study, individuals were systematically excluded from the final cohort analysed in this study based on the following criteria: 1) large focal brain damage (i.e. more than 1/3 of one hemisphere) as stated by an expert in neuroanatomy blinded to the patients' diagnoses; 2) excessive head motion during resting state scaning (i.e. greater than 3mm in translation and/or 3 degrees in rotation); 3) suboptimal segmentation and normalization of images. One additional patient was excluded due to incomplete acquisition. Thus, the final cohort analysed in this study comprised 22 patients (13 males; 17 -70 years; mean time post injury: 13 months; 10 UWS, 11 MCS).
Non-participation	No participants declined participation.
Randomization	No randomisation was present: for the anaesthesia dataset, all participants were run in both conditions (awake and anaesthetised) since this was a repeated measures design. The DOC patients were drawn from the local patient population, and compared with locally recruited healthy controls.

Reporting for specific materials, systems and methods

We require information from authors about some types of materials, experimental systems and methods used in many studies. Here, indicate whether each material, system or method listed is relevant to your study. If you are not sure if a list item applies to your research, read the appropriate section before selecting a response.

Materials & experimental systems

n/a	Involved in the study	
☒	☐	Antibodies
☒	☐	Eukaryotic cell lines
☒	☐	Palaeontology and archaeology
☒	☐	Animals and other organisms
☐	☒	Human research participants
☒	☐	Clinical data
☒	☐	Dual use research of concern

Methods

n/a	Involved in the study	
☒	☐	ChIP-seq
☒	☐	Flow cytometry
☐	☒	MRI-based neuroimaging

Human research participants

Policy information about studies involving human research participants

Population characteristics	See above.
Recruitment	For the anaesthesia study (London, ON dataset) and the healthy controls of the Cambridge dataset, we recruited participants with posters around campus as per ethics protocol. Participants approaches the research team to seek participation, and there were no specific selection biases. Participants were required to be healthy, right-handed, native English speakers with no history of neurological disorders, and no contraindications to MRI scanning.<br>DOC patients were recruited from specialised long-term care centres. To be invited to the study, patients must have had a DOC diagnosis, written informed consent to participation from their legal representative, and were capable of being transported to Addenbrooke's Hospital. The exclusion criteria included any medical condition that made it unsafe for the patient to participate (decision made by clinical personnel blinded to the specific aims of the study) or any reason they are unsuitable to enter the MRI scanner environment (e.g. non-MRI-safe implants), significant pre-existing mental health problems, or insufficient English pre-injury.
Ethics oversight	Ethical approval for testing DOC patients was provided by the National Research Ethics Service (National Health Service, UK; LREC reference 99/391). The Cambridgeshire 2 Research<br>Ethics Committee approved the study (LREC 08/H0308/246) for the healthy controls of the Cambridge dataset. For the anaesthesia dataset, the Health Sciences Research Ethics Board and Psychology Research Ethics Board of Western University (Ontario, Canada) ethically approved the study.

Note that full information on the approval of the study protocol must also be provided in the manuscript.

Magnetic resonance imaging

Experimental design

Design type	Resting-state.
Design specifications	DOC dataset: 10 minutes continuous scan; Cambridge healthy controls: 5:20 minutes continuous scan; anaesthesia dataset: 8 minutes continuous scan.
Behavioral performance measures	In both datasets, lack of behavioural responsiveness was used as an indicator of unconsciousness.<br>For the DOC patients, it was assessed clinically at multiple times over the period of their stay in Addenbrooke's Hospital (Cambridge, UK).<br>For the propofol dataset, failure to perform two computerised tasks (a computerised auditory target-detection task and a memory test of verbal recall) was used to evaluate the level of wakefulness in the anaesthesia condition independently of the assessors, who also evaluated participants' level of behavioural responsiveness based on the Ramsay scale. For the healthy control dataset (Cambridge), behavioural measures unrelated to this study were also collected.

Acquisition

Imaging type(s)	Functional and anatomical and diffusion.
Field strength	3T for all datasets.
Sequence & imaging parameters	Propofol dataset: MRI scanning was performed using a 3-Tesla Siemens Tim Trio scanner (32-channel coil), and 256 functional volumes (echo-planar images, EPI) were collected from each participant, with the following parameters: slices = 33, with 25% inter-slice gap; resolution = 3mm isotropic; TR = 2000ms; TE = 30ms; flip angle = 75 degrees; matrix size = 64x64. The order of acquisition was interleaved, bottom-up. Anatomical scanning was also performed, acquiring a high-resolution T1- weighted volume (32-channel coil, 1mm isotropic voxel size) with a 3D MPRAGE sequence, using the following parameters: TA = 5min, TE = 4.25ms, 240x256 matrix size, 9 degrees FA.<br><br>DOC patients: Resting-state fMRI was acquired for 10 minutes (300 volumes, TR=2000ms) using a Siemens Trio 3T scanner (Erlangen, Germany). Functional images (32 slices) were acquired using an echo planar sequence, with the following parameters: 3 x 3 x 3.75mm resolution, TR = 2000ms, TE = 30ms, 78 degrees FA. Anatomical scanning was also performed, acquiring high-resolution T1-weighted images with an MPRAGE sequence, using the following parameters: TR = 2300ms, TE = 2.47ms, 150 slices, resolution 1 x 1 x 1mm.<br><br>Healthy controls (Cambridge): Resting-state fMRI was acquired for 5:20 minutes (160 volumes, TR=2000ms) using a Siemens Trio 3T scanner (Erlangen, Germany). The acquisition parameters were the same as those for the DOC patients: Functional images (32 slices) were acquired using an echo planar sequence, with the following parameters: 3 x 3 x 3.75mm resolution, TR = 2000ms, TE = 30ms, 78 degrees FA. High-resolution T1-weighted anatomical images were also acquired, using an MPRAGE sequence with the following parameters: TR = 2300ms, TE = 2.47ms, 150 slices, resolution 1 x 1 x 1mm.
Area of acquisition	Whole brain

Diffusion MRI ☒ Used ☐ Not used

Parameters	The DOC patients' data were acquired over the course of several years, and as a result two different diffusion-weighted image acquisition schemes were used. For the first acquisition scheme, we collected 5 sets of 12 non-collinear diffusion-sensitising gradient directions, each set using a different b-value (5 b-values in total) ranging from 340 to 1,590 s/mm2; therefore, a total of 60 diffusion-weighted volumes were acquired for each patient with this acquisition scheme. The second, more recent acquisition scheme included 63 directions with a b-value of 1000 s/mm2; this acquisition scheme was adopted for all remaining DOC patients and also for all healthy controls (Cambridge dataset). No diffusion-weighted imaging was acquired for the propofol dataset.<br>Healthy controls (Cambridge): TR = 8300 ms, TE = 98 ms, matrix size = 96 x 96, 63 slices, slice thickness = 2 mm, no gap, flip angle = 90°, 63 directions with a b-value of 1000 s/mm2.

Preprocessing

Preprocessing software	We used the CONN toolbox version 17f, based on SPM12. The default pipeline was used. The pipeline comprised the following steps: removal of the first five scans, to allow magnetisation to reach steady state; functional realignment and motion correction; slice-timing correction to account for differences in time of acquisition between slices; identification of outlier scans by means of the quality assurance/artifact rejection software ART; spatial normalisation to Montreal Neurological Institute (MNI-152) standard space with 2mm isotropic resampling resolution, using the segmented grey matter image from each volunteer's high-resolution T1-weighted image, together with an a priori grey matter template; spatial smoothing with a Gaussian kernel of 6mm full width at half-maximum (FWHM).<br>Due to the presence of deformations caused by brain injury, rather than relying on automated pipelines, DOC patients' brains were individually preprocessed using SPM12, with visual inspections after each step, and subsequently denoised following the steps outlined above.<br><br>The diffusion data were preprocessed with MRtrix3 tools, following the same pipeline as in our previous work 61,125. After manually removing diffusion-weighted volumes with substantial distortion 54, the pipeline involved the following steps: (i) DWI data denoising by exploiting data redundancy in the PCA domain 126 (dwidenoise command); (ii) Correction for distortions induced by eddy currents and subject motion by registering all DWIs to b0, using FSL's eddy tool (through MRtrix3 dwipreproc command); (iii) rotation of the diffusion gradient vectors to account for subject motion estimated by eddy 127; (iv) b1 field inhomogeneity correction for DWI volumes (dwibiascorrect command); (v) generation of a brain mask through a combination of MRtrix3 dwi2mask and FSL BET commands.<br><br>After preprocessing, the DTI data were reconstructed using the model-free q-space diffeomorphic reconstruction algorithm (QSDR) implemented in DSI Studio (www.dsi-studio.labsolver.org) 128, following our previous work 61,129. Finally, fiber tracking was carried out by means of DSI Studio's own "FACT" deterministic tractography algorithm, requesting 1,000,000 streamlines according to widely adopted parameters 61,129,132–134.
Normalization	Direct normalisation to MNI space (nonlinear) using the segmented grey matter image from each volunteer's high-resolution T1-weighted image, together with an a priori grey matter template.
Normalization template	MNI-152 template.
Noise and artifact removal	The anatomical CompCor method was used, regressing out of the functional data the following confounding effects: the first five principal components attributable to each individual's white matter signal, and the first five components attributable to individual cerebrospinal fluid (CSF) signal; six subject-specific realignment parameters (three translations and three rotations) as well as their first- order temporal derivatives; the nuisance regressors identified by ART; and main effect of scanning condition. Linear detrending was also applied, and the subject-specific denoised BOLD signal timeseries were band-pass filtered to eliminate both low-frequency drift effects and high-frequency noise, thus retaining frequencies between 0.008 and 0.09 Hz. Additionally, to further reduce potential movement artifacts, data underwent despiking using the hyperbolic tangent squashing function from the CONN toolbox 123. This method applies a continuous squashing function to the BOLD signal, rather than utilizing an absolute threshold that would result in cropping any values above that threshold.
Volume censoring	The artifact rejection tool (ART), implemented in the CONN toolbox, was used to identify and regress out outlying volumes, as part of the CompCor denoising procedure described above. The default CONN settings of 5 global signal z-values and 0.9mm were used. For the awake healthy volunteers, this resulted in an average of 1% of volumes being identified as outliers; in the anaesthetised condition, a mean of 10% of volumes were identified as artifactual. For the DOC patients, the mean percentage of volumes identified as artifacts was 11%.

Statistical modeling & inference

Model type and settings	To test the effects of various procedures on the dynamics simulated from the DMF model, statistical differences were evaluated for significance at the standard alpha level of 0.05 (two-sided), using permutation-based between-subjects t-tests on the distributions of KS-distance values obtained from N=100 simulations from the corresponding models being compared. Mean and standard error of the mean of the data are displayed in the Figures. Tables S1-S4 report the test results. Effect sizes were estimated as Cohen's d (standardised difference of means).
Effect(s) tested	We evaluated goodness-of-fit in terms of the KS-distance between simulated and empirical dynamics, and tested whether the KS distance to data from unconscious individuals would decrease for our GABA-informed models; we also tested whether different connectome perturbations would increase the relative fit to unconscious vs conscious individuals

Specify type of analysis: ☒ Whole brain ☐ ROI-based ☐ Both

Statistic type for inference<br>(See Eklund et al. 2016)	The Kolmogorov-Smirnov distance was used to quantify the goodness-of-fit measure of simulated brain dynamics obtained from a whole-brain computational model (dynamic mean field model).
Correction	Not applicable.

Models & analysis

n/a	Involved in the study
☐	☒ Functional and/or effective connectivity
☒	☐ Graph analysis
☐	☒ Multivariate modeling or predictive analysis

Functional and/or effective connectivity

Following Deco et al., (2018), functional connectivity dynamics (FCD) were quantified in terms of Pearson correlation between regional BOLD timeseries, computed within a sliding window of 30 TRs with increments of 3 TRs. Subsequently, the resulting matrices of functional connectivity at times tx and ty were themselves correlated, for each pair of timepoints tx and ty, thereby obtaining an FCD matrix of time-versus-time correlations. Thus, each entry in the FCD matrix represents the similarity between functional connectivity patterns at different points in time.

Multivariate modeling and predictive analysis

Whole-brain spontaneous brain activity (as quantified using blood oxygen level dependent (BOLD) signal data from functional MRI) was simulated using a neurobiologically realistic Dynamic Mean Field (DMF) model. This model has one free parameter, the global coupling G. We fitted the model to empirical functional connectivity dynamics (FCD) (Deco et al., 2018), using the Kolmogorov-Smirnov distance to compare the histograms of empirical and simulated FCD values (obtained from the upper triangular FCD matrix), to find the G parameter that results in the best match between empirical and simulated functional connectivity dynamics. The same KS-distance was also used as the goodness-of-fit measure to quantify the similarity between empirical and simulated macroscale brain activity. To find the value of G that generates simulations whose FCD best match empirical FCD, we generated 100 simulations for each value of G between 0.1 and 2.5, using increments of 0.1. For each simulation at each value of G, we computed the KS distance between empirical (group-wise) and simulated FCD. Finally, we set the model's G parameter to the value that minimised the mean KS distance - corresponding to the model that is best capable of simulating the temporal dynamics of functional connectivity observed in the healthy human brain at rest.
